# A new mechanism for efficient hydrocarbon electro-extraction from *Botryococcus braunii*

**DOI:** 10.1186/s13068-017-0724-1

**Published:** 2017-02-13

**Authors:** Alexis Guionet, Bahareh Hosseini, Justin Teissié, Hidenori Akiyama, Hamid Hosseini

**Affiliations:** 1 Bioelectrics Department, Institute of Pulsed Power Science, Kumamoto University, 2-39-1 Kurokami, Kumamoto, 860-8555 Japan; 2 Graduate School of Science and Technology, Kumamoto University, Kumamoto, Japan; 3 Institute of Pharmacology and Structural Biology, University Paul Sabatier, 205 Route de Narbonne, 31077 Toulouse, France

**Keywords:** Green energy, Biofuel, Hydrocarbon, Extraction, *Botryococcus braunii* kützing, Nanosecond pulsed electric field (nsPEF)

## Abstract

**Background:**

Recent understanding that specific algae have high hydrocarbon production potential has attracted considerable attention. *Botryococcus braunii* is a microalga with an extracellular hydrocarbon matrix, which makes it an appropriate green energy source.

**Results:**

This study focuses on extracting oil from the microalgae matrix rather than the cells, eliminating the need for an excessive electric field to create electro-permeabilization. In such a way, technical limitations due to high extraction energy and cost can be overcome. Here, nanosecond pulsed electric fields (nsPEF) with 80 ns duration and 20–65 kV/cm electric fields were applied. To understand the extraction mechanism, the structure of the algae was accurately studied under fluorescence microscope; extraction was quantified using image analysis; quality of extraction was examined by thin-layer chromatography (TLC); and the cell/matrix separation was observed real-time under a microscope during nsPEF application. Furthermore, optimization was carried out by screening values of electric fields, pulse repetition frequencies, and energy spent.

**Conclusions:**

The results offer a novel method applicable for fast and continues hydrocarbon extraction process at low energy cost.

**Electronic supplementary material:**

The online version of this article (doi:10.1186/s13068-017-0724-1) contains supplementary material, which is available to authorized users.

## Background

Biodiesel from algae is a promising solution in the field of green energy. While energy derived from petroleum generates a huge carbon footprint, biodiesel generates an identical amount of carbon as is captured by the feed algae production [1]. Indeed, while growing, algae captures carbon from atmospheric CO_2_ for diverse metabolic activity, especially hydrocarbon formation. All carbon used for fatty acid formation comes from atmospheric CO_2_, one reason why biodiesel has grown increasingly important in the new field of bioenergy. Additionally, improving production efficiency will lead to lower cost. Currently, biodiesel is generally derived from plant crops, but a more promising method is by using microalgae [2, 3]. Some microalgae species are able to produce high-quantity hydrocarbons which can be rendered to biogasoline. Moreover, for growing algae, arable areas are not required and production costs can be low [4]. However, regardless of whether the feedstock is microalgae or plant crops, hydrocarbon extraction requires energy.

Pulsed electric field (PEF) is anticipated as a promising method for hydrocarbon extraction from microalgae as it is able to permeabilize membranes and to weaken walls of cells [5–8]. Once permeabilized, some intracellular elements may be extracted from cells; protein, for example, can be extracted from intracellular algae using PEF [9–11]. Regarding pulse length, nanosecond pulsed electric fields (nsPEF) have been shown to be more energy efficient than millisecond or microsecond pulsed electric fields (msPEF or µsPEF); as nsPEF pulses are shorter, they generally consume less energy even when applied voltages are higher [12].

*Botryococcus braunii* is a green planktonic microalga, which has the specificity to build extracellular networks of polysaccharides and hydrocarbons as colonial matrixes [13]. These both allow cells to adhere as colonies and to float on water surfaces, because hydrocarbons have lower density and are lighter than water [14]. Such matrixes contain over 99% C33 and C34 compounds [15]. These long chain hydrocarbons can be hydrocracked to provide gasoline and fuel [16]. Between 25 and 40% of the oil contained in these matrixes can generally be extracted from dry weight [17], with up to 86% reported under optimal conditions [18]. However, oil extraction from an algae cake obtained by drying processes is mostly destructive and pricey.

Several research groups have noted the difficulty of extracting hydrocarbons from fast-growing microalgae, e.g., *Chlorella vulgaris* and *Nannochloropsis* sp. In these types of microalgae, hydrocarbons are produced inside the cell cytoplasm [19], generally requiring alternative extraction methods [20–24]. On the other hand, hydrocarbons stocked in the matrix of *Botryococcus braunii* have already been secreted out of cells [15], making them easier to access.

The goal of this study is to verify a novel method for fast and efficient hydrocarbon extraction from microalgae. Here, we show that hydrocarbons of *Botryococcus braunii* are more efficiently extractable by nsPEF. Screening various conditions allowed us to set parameters for rapid and energy-efficient extraction. Furthermore, we suggest a new continuous extraction method to allow the *Botryococcus braunii* culture to restart directly after oil and polysaccharides extraction. Such system is adaptable as an industrial oil and polysaccharide extraction process at low energy cost.

## Results

### nsPEF profile

**Fig. 1:**
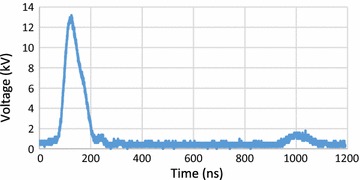
Pulse profile using 10 cuvettes in parallel with main pulse and a negligible spike at 0.9 μs

Figure 1 shows the voltage waveform of the nsPEF with a pulse duration of 80 ns and voltage of 12.8 kV. There was a small unwanted spike of around 12% of maximal voltage at 0.9 µs after the pulse, which, under our experimental conditions, generated an electric field near 7.5 kV/cm, insufficient to have any impact on extraction.

### Fluorescence microscopy of *Botryococcus braunii* colony

**Fig. 2:**
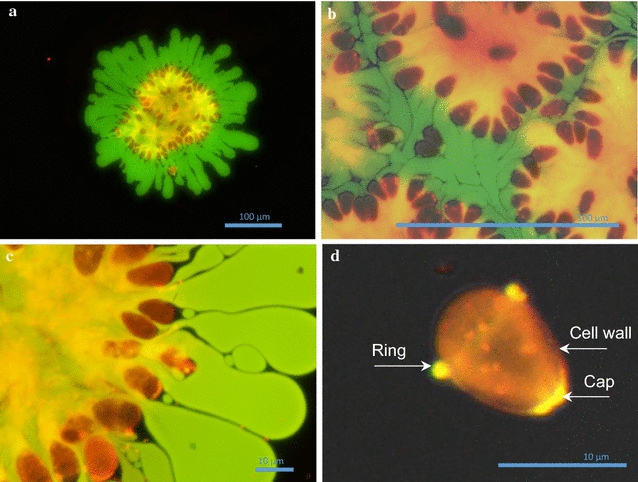
Fluorescent images of hydrocarbon and polysaccharide repartition on algae colonies. **a** Colony full of cells (*red*) with polysaccharides (*yellow*) and hydrocarbons (*green*) leaving the colony (×20); **b** parts of colony tightly packed together (×60), merge of white light with filters 1 and 2; **c** colony full of cells (*red*) with dichotomial ramification of polysaccharides (*yellow*) and hydrocarbons leaving the colony (*green*) (×100). **d** Single cell, merge of white light with filters 1 and 2 (×100 and numerically magnified)

Protocol for fluorescence microscopy is described in the Additional file 1: Section 1-3). Without nsPEF application, due to pressure of the cover glass, hydrocarbons were progressively expended from colonies (Fig. 2a–c). Despite this, we could observe polysaccharides linked to basal parts of each alga cell by dichotomial ramification, and hydrocarbon inside the colony, with cells surrounding the surface of the colonies (Fig. 2a, c).

In Fig. 2d, cell walls colored in red, from where a ring on the upper part and a cap on basal part, produce both hydrocarbons and polysaccharides, to form the matrix. *Botryococcus braunii* is known to be able to form colonies with sizes up to 1 mm, starting from a single cell by the secretion of a matrix containing hydrocarbons [25]. Our observations of colonies and of single cells suggest that the ring on the upper and the cap on the basal parts secrete the matrix from the single cell stage and continue to do so during cell division. This constitutes a kind of anchorage, allowing each cell to retain basal part inside the colony and apical part outside the colony during its development.

The observation (Fig. 2a, c) is important, as it shows that pressing the floating multicellular assembly can yield to hydrocarbon extraction. This in part may explain the nsPEF extraction, if we consider that pressure jumps are induced by each single PEF pulse [26]. While exerting pressure can facilitate a partial hydrocarbon extraction [27], after pressing, the cells are still adhered to the matrix holding considerable amount of hydrocarbons (Fig. 2b, c). This refers to additional mechanisms involved during the nsPEF application, which can promote further extraction and is subject of the next sections.

### Microscopic observation during nsPEF treatment

**Fig. 3:**
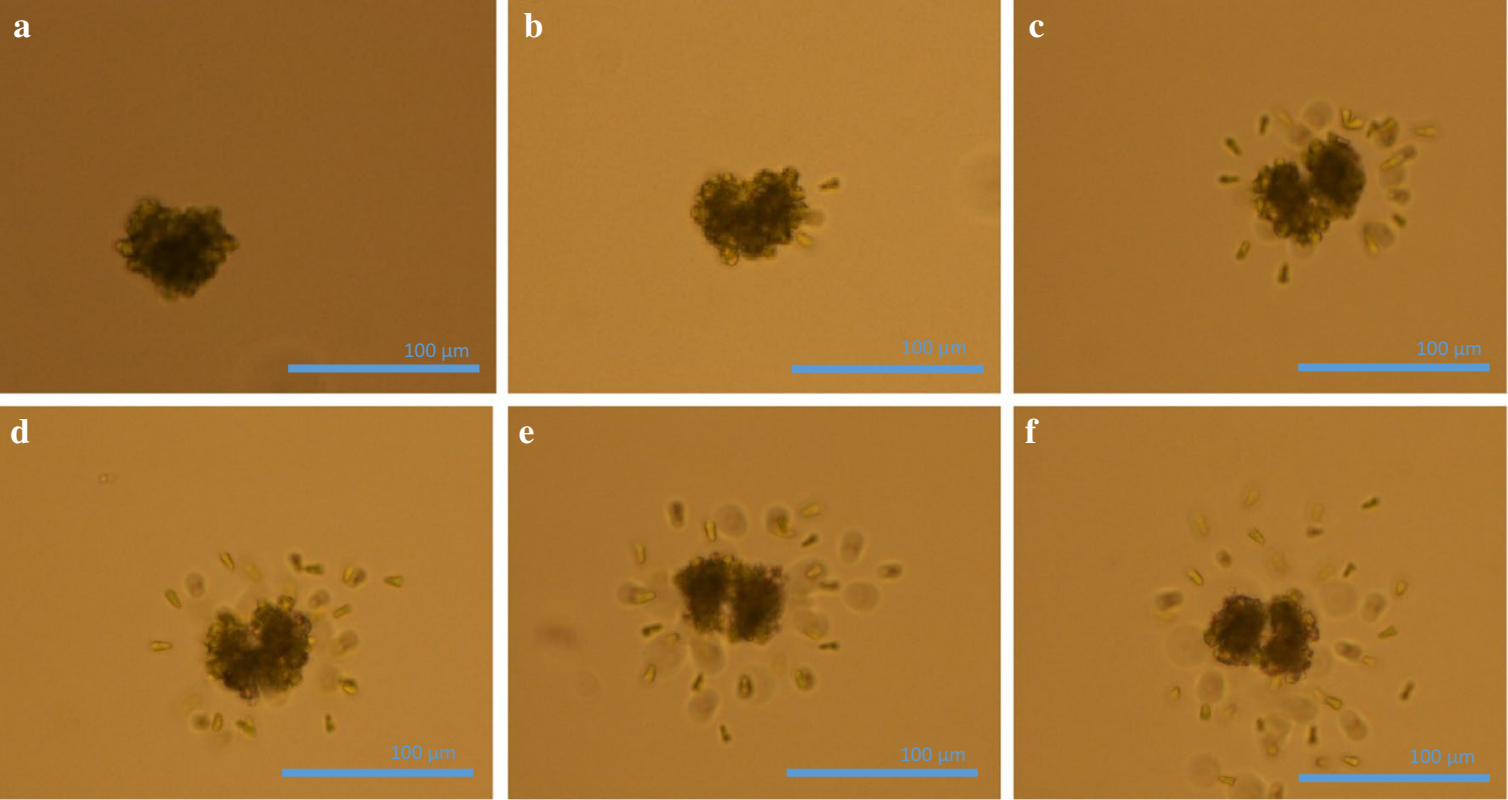
Real-time microscopic observation during treatment (500 pulses, 10 Hz, and 144 kV/cm) (magnification ×20). **a** Before treatment; **b** after 200 pulses at 20 s; **c** after 400 pulses at 40 s; **d** after 60 s; **e** after 100 s; **f** after 500 s

Microalgae were submitted to trains of nsPEF and were observed real-time under a microscope using an electrode assembly (see “Methods” section, Fig. 9). Figure 3 shows selected images for a colony, performed during application of 500 pulses with 10 Hz repletion rate and electric field of 144 kV/cm. The associated color movies taken with the microscope are shown in the “Additional file 2” (treated with nsPEFs) and “Additional file 3” (sham treated), and are described in Additional file 1: Section 2-2).

The images clearly show cells leaving the colony when PEF are applied with consequential separation from the matrix. The microscopic images are consistent with our macroscopic observations (refer to Additional file 1: Section 2-3). The phenomenon starts few seconds after treatment commences and continues for several minutes after treatment ends; its intensity depends on electric field amplitude and applied energy (for comparison of different electric fields, refer to Additional file 1: Section 2-1). This behavior of ‘cell hatching,’ where many cells suddenly leave their holding structure and sedimenting, is a new consequence to the already long list of biological effects of electric field pulses.

### Voltage effects on hydrocarbon extraction

The dissociation effect on the multicellular assembly was clearly affecting the extracellular matrix. This was indeed confirmed by the analysis of the supernatant of pulsed floating microalgae obtained with the macroscopic approach on pulsing cuvettes (see Additional file 1: Section 2-3). Hydrocarbons were electro-extracted as shown by the fluorescence assay (see Additional file 1: Section 2-2).

**Fig. 4:**
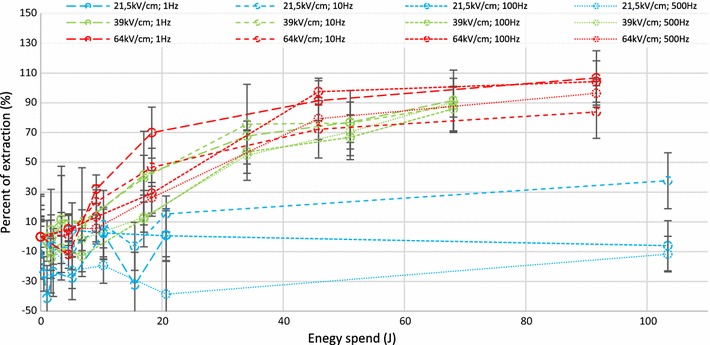
Percent of extraction according to spent energy (0.9 ml of algae), at various electric fields and pulse repetition frequencies

To study the influence of voltage amplitude, three values of 21.5, 39, and 64 kV/cm electric fields were used. The results are summarized in Fig. 4. The spent energy for 2 cuvettes, both filled with 450 µl algae, was considered. Efficiency of electro-extraction was determined using supernatant color analyses. Various frequencies (pulse repetition rates) of 1, 10, 100, and 500 Hz were utilized. Data were compared according to energy spent, which depended on shot number and electric field (voltage). The supernatant was confirmed using TLC to be composed of hydrocarbons, more precisely polar lipids (see Additional file 1: Section 2-4, Figure S5).

Figure 4 shows that 21.5 kV/cm electric field is not sufficient to extract hydrocarbons even with high energy expenditure. Experiments at 39 kV/cm and 64 kV/cm show approximately the same efficiency: 50% of extraction after applying approximately 25 J (27.8 J/ml) and 80% after 50 J (55.6 J/ml), while 64 kV/cm shows a higher rate of efficiency increase at lower energies. The results give evidence that there is an electric field threshold value under which extraction is not possible. The extraction efficiency is not sensitive to the total energy for energies above 50 J (55.6 J/ml). At lower energy levels, the extraction was controlled by the field strength. In Fig. 4, the results are not sensitive to pulse repetition frequency as well; for each electric field, the observations are the same for different frequencies, meaning that it is possible to use fast treatment (i.e., a high shot number applied in a short time) to extract a high amount of hydrocarbons, especially if a flow treatment process for industrial scale would be adapted [9, 10].

### Experiment with homogenous energy

**Fig. 5:**
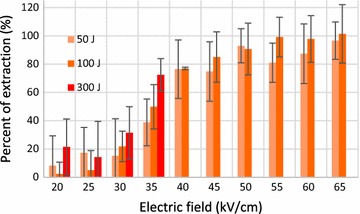
Percent of hydrocarbon extraction according to electric field, at constant energy consumptions

To compile comprehensive data, a series of three experiments were conducted under constant energies; the results are shown in Fig. 5. The shot numbers were chosen depending on the electric field to ensure a constant energy. All experiments were carried out at 10 Hz frequency.

As the thermal effect was not negligible above 35 kV/cm at 300 J (333.3 J/ml) (measured temperature increase was over 5 °C), the experiments were not conducted under those conditions. Full extraction was reached with sufficient electric fields higher than 40 kV/cm. The experiments confirm that 25 kV/cm was insufficient for hydrocarbon extraction even when a large amount of energy was spent. Consequently, augmentation of energy expenditure only displays a positive effect on the extraction when the electric filed is between 30 and 45 kV/cm, though the differences are not significant.

In Fig. 5, of interest is that using two times more energy enables only 10% increase in extraction, e.g., at 45 kV/cm, 50 J, corresponding to 1100 shots for a duration of 110 s, is sufficient for almost 75% extraction; while twice the energy (100 J, corresponding to 2200 shots for a duration of 220 s) results in 85%. Optimal conditions for oil extraction appears to be 50 J (55.6 J/ml) at 50 kV/cm. Therefore, on a large-scale treatment flow system (Fig. 7), the most efficient extraction can be obtained at 50 kV/cm of electric field and 55.6 J/ml of energy per algae volume passing through the flow system.

**Fig. 6:**
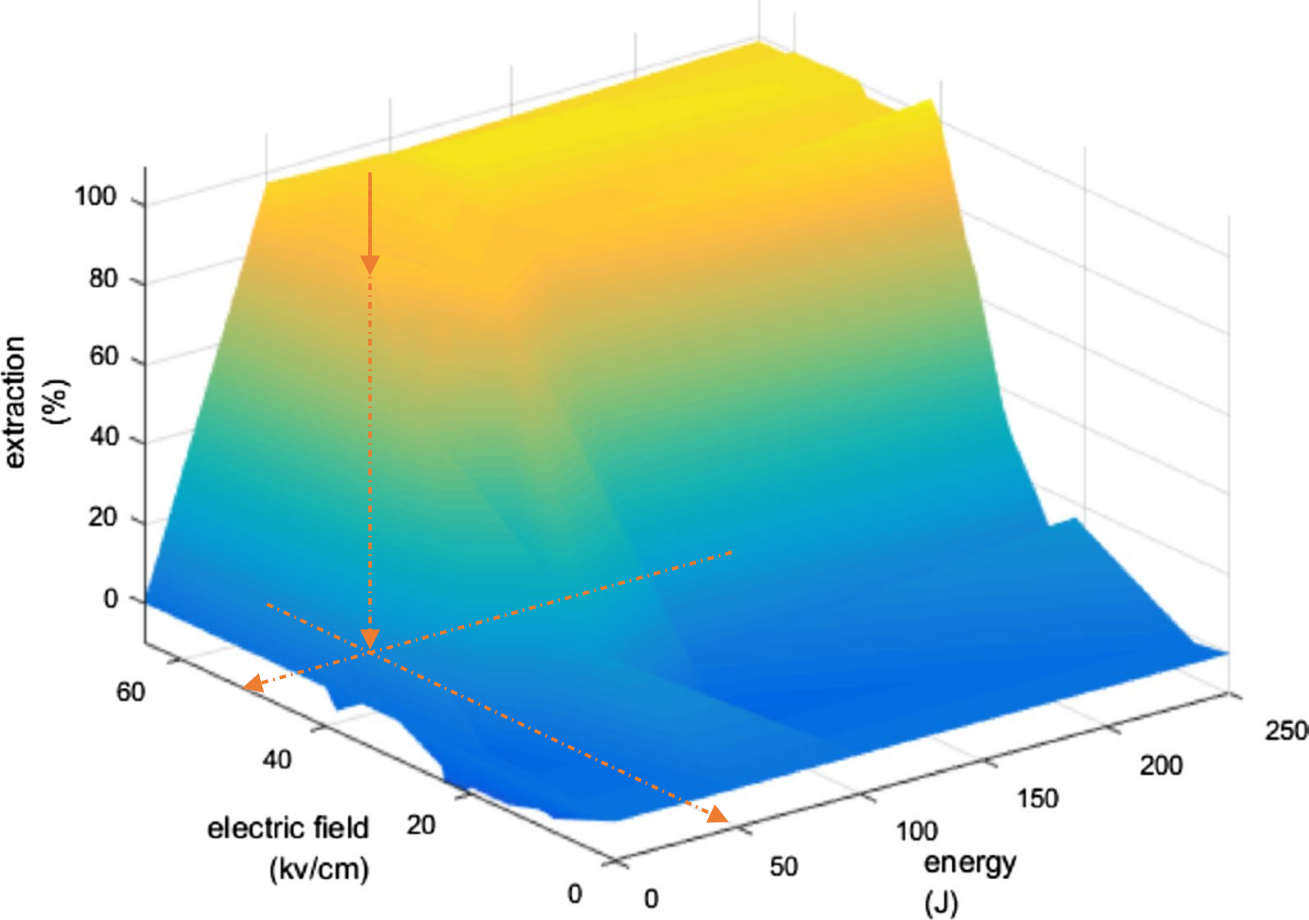
Efficiency of extraction according to energy spent and the electric field. Graphic is drawn by bilinear interpolation

Hydrocarbon extraction using nsPEF is a multi-factorial phenomenon which depends on three interrelated variables: the electric field, pulse number, and energy spent; this is illustrated in a 3D graph in Fig. 6. The graph represents all experimental data of extraction percentage obtained at 10 Hz pulse repetition rate and was drawn by bilinear interpolation.

## Discussion and conclusions

In this study, various voltages, frequencies, and pulse numbers were applied to determine the extraction efficiency based on the energy spent. We also elucidated how our extraction system functions. Combining macroscopic and microscopic observations showed, under a sufficient electric field, the matrix and cells were separated, and that matrix was composed largely of hydrocarbons.

It has been proved that electric field effect on biologic material sometime come from electrostriction [28] or pressure wave [29] induced by the potential difference generated by PEF. In this respect, two hypotheses can be formulated to explain mechanism behind the extraction: (1) The retaining wall ruptures—due to movement of charged molecules, electrostriction, or pressure wave—allow cells to naturally leave the colony. (2) Polysaccharides secreted by cells and solubilized in the oily matrix are detached from cells—due to movement of charged molecules, electrostriction, or pressure wave—and having lost their anchor, cells can no longer adhere to the colony. As the retaining wall was not often observed, it is not possible to confirm the first hypothesis. Conversely, as we could see evidence of polysaccharides inside the oily matrix (Fig. 2a–c), and as detached cells leaving the colony are mostly free of oil/polysaccharides except for the cell wall (Fig. 2d; Additional file 1: Section 2-2, movies of Additional files 2, 3), there is clearly a separation between cells and oil/polysaccharide of the matrix when nsPEF are applied, supporting the second hypothesis as prevailing mechanism.

Another hypothesis to explain the mechanism behind the electric field induced cell detachment can also be considered: (3) The cells in the colony might be linked together by actin filaments [30]. Exposure to nsPEF destroys actin, loosening the link between the cells, causing them to leave the colony. Indeed, in plant cells, during phragmoplasme formation, plasmodesmata are built in such a way to connect cytoplasm and actin filaments even after the end of cell division [30]. Berghöfer et al. [31] showed that nsPEF trigger actin responses in plant cells. Positive effect of PEF on actin disassembling was also shown [32]. Further investigations should focus on actin filaments observation to validate this hypothesis, as plasmodesmata might not exist for *Botryococcus braunii* cells and actin might be restricted into the cytoplasm.

The extraction mechanism here is completely different from electro-extraction of other microalgae species. Other unicellular algae species, which also produce a large amount of oil, do not secrete a matrix. In those cases, the electric field must destroy the plasma membrane—or at least cause poration—in order to liberate hydrocarbon vesicles, which require higher amount of electric energy. Goettel [21] showed that 1 MJ is required to rupture cells of 1 kg of dry weight algae (*Auxenochlorella protothecoides)* from a suspension of near 100 g of dry weight algae per kg of suspension. The author also showed that the concentration of algae suspension did not affect efficiency [21]. In that case, 0.1 MJ was required to treat approximately one liter of algae suspension (=100 J/ml). Our treatment using two electroporation cuvettes of 450 µl (=0.9 ml) enabled extraction using 50 J (55.6 J/ml), almost with two times higher efficiency. The matrix also contains polysaccharides; therefore, the supernatant is not pure hydrocarbon and thus needs further purification. Polysaccharides hold high interest in the field of green energy as they may be used to produce bioethanol [33]; however, polysaccharides extract would be in low concentration as high percentage of the dry mass of *Botryococcus braunii* consists of hydrocarbons [34].

According to our screening, the best performance may be achieved at 50 kV/cm, where the lowest energy consumption was needed for extraction. Higher energy spent only marginally improves extraction, and a higher electric field would not be necessary. Also shown was that frequencies from 1 to 500 Hz have no effect on efficiency, meaning that treatment can be rapid and thus adaptable as an industrial process.

**Fig. 7:**
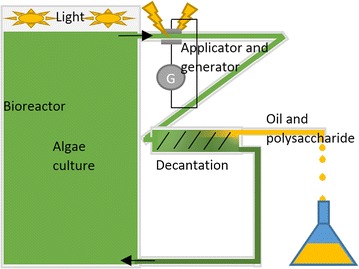
Possible assembly for industrial continuous production of lipids and polysaccharides

As our method with nsPEF allows use of electric fields and energies lower than the cell irreversible membrane damage threshold [35, 36], a high portion of the algae cells may survive during the extraction. This is an important advantage; as such, it may be considered an ‘in vivo extraction’ method (Fig. 7).

Current oil extraction processes are mostly destructive; after the culture reaches a stationary state, oil is extracted from an algae cake obtained by drying [37, 38]. Conversely, our method may allow the culture to restart directly after oil and polysaccharide extraction, like chemical extraction method [39]. As such, our system is adaptable as a continuous oil and polysaccharide extraction process at low energy cost. In the flow treatment process, electric field application to treat large volumes was examined for bacterial eradication and protein extraction from microalgae [9, 10, 12, 40]. In particular, it is possible to design a system using a derivation to separate algae culture from a bioreactor, treat it by nsPEF, extract oil and polysaccharide by in-line decantation (lamellar decanter might help to lead single cells on the bottom part and matrix on the upper part), and then return the medium and surviving algae to the bioreactor, as shown in Fig. 7. In such a system, production of oil and polysaccharide might be faster than in a batch system, as the culture would not have to restart from zero. After treatment, cells can directly restart to grow and divide to produce more oil and polysaccharide (Fig. 7). This type of system might also minimize the initial as well as running cost, because the separation of cells and extract can be done rapidly by simple decantation, thus requiring little energy and space.

## Methods

### *Botryococcus braunii* growth

A frozen preserved strain of *Botryococcus braunii* Kützing race B (NIES836) from the National Institute for Environmental Studies (NIES) was used to raise the pre-culture, 30 ml of which was incubated at 21 °C with 200 ml of AF-6 medium under agitation (40 rpm), permanent light (photon flux density = 20 µmol/m^2^/s^1^), and air containing 2% CO_2_ supply. The batch was cultivated in pre-culture and culture steps. The culture medium was changed and renewed between the pre-culture and culture steps to provide additional nutrients for the growth. The culture was grown for about 100–130 days to reach to the stationary phase. The inoculum was standardized, and the initial concentration was fixed at 10^6^ colonies/ml. Colony organizations were observed under a fluorescence microscope (Nikon, Eclipse Ti-U) using oil and polysaccharides stain (for more information, refer to Additional file 1: Section 1-3).

### Pulsed electric field application

**Fig. 8:**
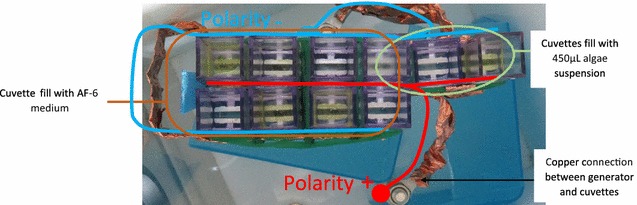
Photo of connection between cuvettes under treatment with nsPEF

The generator used was a magnetic pulse compression (MPC) modulator [41, 42] (nsBioPEFs, Fusiontech, Japan) with 30 Ω internal resistance. Eight cuvettes containing AF-6 media (350 µs/cm) of 286 Ω resistance and 2-mm gap electroporation (Molecular Bio-Product) were connected in parallel. Except for experiments requiring large volumes, these cuvettes were only used for impedance matching. Two cuvettes were connected in parallel (total 10 parallel cuvettes) and each filled with 450 µl of the microalgae culture for experiments, as shown in Fig. 8.

**Fig. 9:**
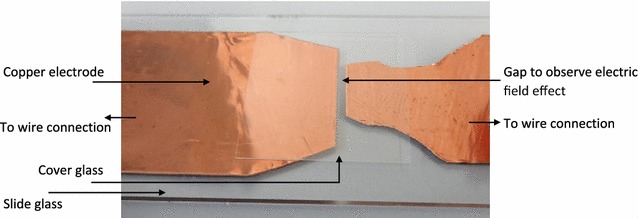
The composition of the test section used to apply nsPEF under microscope

Figure 9 shows a test section made for real-time observations under the microscope. The test section has a gap distance of 0.9 mm. It consists of two 0.2-mm thickness copper plate electrodes, a slide, and a cover glass. It was connected in parallel with the cuvettes of Fig. 8; due to its high resistance, the same voltage waveform as Fig. 1 was recorded for the micro-gap.

Voltage was recorded by a Tektronix P6015A (1000×, 3.0 pF, 100 MΩ) voltage probe. The oscilloscope used was a Tektronix DPO-4104 digital phosphor oscilloscope. Current was recorded using the device’s internal probe.

### Quantification of hydrocarbon extraction

After pulse treatment, the suspension in the electroporation cuvettes was homogenized, and 880 µl was taken into a micro-tube. Micro-tubes were incubated for 1 h at room temperature, centrifuged for 15 min at 2000*g* (centrifuge separated the colonies to remain at the surface and single cells to be collected as pellet), and then photographed. For all series of experiments, photos were taken under identical light conditions and in front of a white background. Samples from each experiment series were recorded in the same photographic set. Photos were analyzed with the open source software “ImageJ” [43] using the plugin called RGB_profiler [44]. For each sample, red, green, and blue values of the sample supernatant were recorded, and green over red values were calculated. Color was evaluated from about 300 pixels, taken from right all the way to the left part of each sample’s photograph. The cylindrical micro-tubes had lower light exposure on the left and right sides compared to the middle, causing deviations from the mean value for each sample evaluation.

Green over red value from supernatant of the control sample was used as a value of 0% of extraction, and 1 was referred to 100%. According to RGB encoding, when red and green value are equal (red over green = 1), it means that color of the pixel is a shade of yellow, corresponding to no green color. When supernatant was no more green, it contained no alga cell (no more presence of chlorophyll), corresponding to total extraction.

As hydrocarbons are lighter than water, they appeared as supernatants, and the quality of extraction was characterized by the color of the supernatant. Their color progressively turned from green to yellow due to separation between cells (green) and oily matrix (yellow/brown) (for details, refer to Additional file 1: Sections 2-3 and 2-5). No solvents and a low amount of algae were required, enabling a rapid screening of several conditions. To validate the evaluation, solvents were used to purify hydrocarbon of nsPEF treated samples, and those were compared with a thermally treated sample. Hydrocarbon composition was then analyzed by thin-layer chromatography (TLC) (refer to Additional file 1: Sections 1-2 and 2-4).

## Additional files

**Additional file 1.** Additional materials and methods and results; including Figures S1 to S7 and Table S1.

**Additional file 2.** Algae colony treated with 64 kV/cm nsPEF.

**Additional file 3.** Control algae colony (sham treated).

## Abbreviations

C33: lipids with chain carbon with 33 carbons
C34: lipids with chain carbon with 34 carbons
CO2: carbon dioxide
MPC: magnetic pulse compression
µsPEF: microsecond pulsed electric field
msPEF: millisecond pulsed electric field
*Nannochloropsis* sp.: *Nannochloropsis species*
NIES: National Institute for Environmental Studies
nsPEF: nanosecond pulsed electric field
PEF: pulsed electric field
RGB: red green blue color model read by image
RPM: revolutions per minute
TLC: thin-layer chromatography
