# Supplementary material for: A new mechanism for efficient hydrocarbon electro-extraction from Botryococcus braunii
Source: Biotechnol Biofuels. 2017 Feb 13;10:39. doi: 10.1186/s13068-017-0724-1 (PMC5307828; doi:10.1186/s13068-017-0724-1)
Supplement: Supplementary file 1 — Additional file 1. Additional materials and methods and results; including Figures S1 to S7 and Table S1. [file 13068_2017_724_MOESM1_ESM.docx]

Supplementary Material for

**A new mechanism for efficient hydrocarbon electro-extraction from *Botryococcus braunii***

Alexis Guionet ^1^, Bahareh Hosseini ^2^, Justin Teissie ^3^, Hidenori Akiyama ^1,2^,

Hamid Hosseini ^1,2,^*

^1^ Bioelectrics Department, Institute of Pulsed Power Science, Kumamoto University, 2-39-1 Kurokami, Kumamoto, 860-8555, Japan

^2^ Graduate School of Science and Technology, Kumamoto University, Japan

^3^ Institute of Pharmacology and Structural Biology, University Paul Sabatier, 205 route de Narbonne, F-31077 Toulouse, France

*Correspondence: [hosseini@kumamoto-u.ac.jp](mailto:hosseini@kumamoto-u.ac.jp)

This file includes:

1. Materials and Methods
   1. Parallel connection of cuvettes to the pulsed power generator
   2. Identifying lipids by thin layer chromatography (TLC)
   3. Sample preparation for fluorescence microscopy
2. Results
   1. Real-time observation during nsPEF treatment under microscope
   2. Comparison of the treated and sham treated colonies
   3. Macroscopic evaluation
   4. Thin layer chromatography
   5. Image analysis
   6. Schematic representation of *botryococcus braunii* growth

List of supplementary material Figures:

1. Fig. S1
2. Fig. S2
3. Fig. S3
4. Fig. S4
5. Fig. S5
6. Fig. S6
7. Fig. S7

List of supplementary material Table:

1. Table S1

List of Supplementary Movies:

1. Movie S1 Algae colony treated with 64 kV/cm nsPEF "Additional file 2"
2. Movie S2 Control algae colony (sham treated) "Additional file 3"

# Materials and Methods

## Parallel connection of cuvettes to the pulsed power generator

A schematic diagram representing the connection between the electroporation cuvettes and the nanosecond pulsed power generator is shown in Fig. S1.

+

-

Electroporation cuvette

Copper connection to generator positive side

Cuvette electrode

Copper connection to generator negative side

***Fig. S1.*** *Schematic diagram of 10-cuvette applicator connections*

Energy spend per pulse on 2 cuvettes was calculated based on Ohm’s law, as voltage current waveforms of the generator showed that Ohm’s law was valid [1,2]. Each cuvette was filled with 450 μl of the microalgae solution.

## Identifying lipids by thin layer chromatography (TLC)

Oil extraction by cyclohexane for heat-treated algae

Around 40 mL of untreated culture was centrifuged for 15 min at 2,000 g, then 10 mL of supernatant was taken and inserted to a test tube. After a second centrifuge, 5 mL of supernatant, which contained of colonies, was kept. The concentrated algae sample was then heated to 80°^C^ over 30 min so as to destroy cells membrane. 5 mL of cyclohexane was added to the test tube and vigorously shaken for 10 min using a vortex. After incubation for 2 h at room temperature and centrifuge, the organic phase was carefully extracted. Cyclohexane then was evaporated under an extractor fan to keep only oil.

Oil extraction by solvent from sham control and nsPEF treated algae

Total of 100 mL of the algae culture was used. In each treatment, 4.5 mL was treated with 64 kV/cm electric field, 500 pulses at 10 Hz using 10 cuvettes connected in parallel. Treatment was repeated to obtain 50 mL of treated algae. 50 mL of treated and 50 mL of untreated (sham control) cultures were centrifuged for 15 min at 2000 g (separate tubes). After incubation overnight at room temperature, 15 mL of media was removed to increase algae concentration. 10 mL of hexane: isopropanol 3:2 solvent was added and vigorously shaken using a vortex. After incubation for 2 h at room temperature, the supernatant (organic phase) was carefully extracted without extracting any volume of the aqueous phase. After five days of evaporation under an extractor fan, less than 500 µL remained in the tube. Both the sham control and treated samples at this stage were used for thin layer chromatography.

Pure sample for comparisons

To identify lipids extracted, different types of pure lipids underwent TLC, their retention factors were then compared after migration. Pure lipids used were: phosphatidylcholine (L-α-Phosphatidylcholine P3556- 25 mg, Sigma Aldrich) as phospholipid, tristearin (Glyceryl tristearate 69498-250G-F, Sigma Aldrich) as triglycerides, and squalene (S3626- 10 ml, Sigma Aldrich) as triterpene close to botryococcene lipid.

Two mixtures of solvents were used: one for polar lipids, chloroform : acetone : methanol : acetic acid : water, with respective volumes of: 50 : 20 : 10 : 10 : 5; the other for non-polar (apolar) lipids, hexane : diethyl ether : acetic acid, with respective volumes of: 80 : 20 : 1.

Thin layer chromatography (TLC) procedure

The thin layer used was an inorganic silica gel matrix (Analtech TLC Uniplates) sized 10 x 20, separated in the middle to get two plates of 10 x 10. A line was drawn 1.5 cm from the bottom of the uniplate. At each centimeter from the left, a small droplet from different component was placed on the line (a new capillary tubing for each component). Cyclohexane was used to solubilize solid elements. The uniplate was checked under UV before migration. If any extract did not appear clearly under UV, more droplets were added to achieve sufficient concentration; this was especially needed for the sham control (untreated). Drop on spot 2: oil extracted with hexane:isopropanol (3:2) after nsPEF; spot 3: oil extracted by cyclohexane and treatment at 80°C; spot 4: phosphatidylcholine; spot 5: tristearine; spot 6: Squalene; spot 7: oil extracted with hexane:isopropanol (3:2) from sham control. Spot 1 was blank to avoid any boarder effect. All components were dried on the uniplate. Solvent mixtures were placed in two closed large glass container (less than 1cm high). TLCs were kept in the container to allow the solvents to migrate for 15 min. The uniplates were then removed, allowed to dry, and revealed under UV light both directly and also after spraying with Rodmine-B (1 g/L).

## 1.3 Sample preparation for fluorescence microscopy

Nile red (Wako Pure Chemical Industries, Ltd., Japan, Ref. No. 144-08811) was used to stain lipids [3]. Periodic acid was used with propidium iodide (PI) as Schiff reagent to stain polysaccharide as done by Weiss *et al* 2012 [4]. 30 µl of Periodic acid (10%) was added to 270 µl of culture of *Botyococcus braunii*. After incubation in the dark for 30 min at room temperature, 1 ml of distillated water was mixed with the sample to stop the reaction. After centrifuging at 10,000 g for 30 s, by pipetting the liquid under supernatant excess solution was removed (colonies were kept). The washing process was repeated for three times. Following the final washing, 30 µl of phosphate buffer saline (PBS) was used to re-suspend the sample. 1 µl of PI (1 mg/ml) and 1 µl of Nile red (0.15 mg/ml) were added to the sample and incubated in the dark at room temperature for 20 min. After rinsing 3 times with 1 ml of distillated water (centrifuging at 10,000 g for 30 s), 10 µl of sample was placed between slide and cover glasses and was observed under fluorescent microscope (Nikon Eclipse Ti-U Inverted Microscope, equipped with a Nikon Digital Sight DS-Fi1 camera).

***Table S1.*** *Excitation/emission of filters and dyes (no match: - ; weak match: +; strong match: ++).*

|  |  |  | Matching with filter 1 | | Matching with filter 2 | |
| --- | --- | --- | --- | --- | --- | --- |
| dye | excitation | emission | Excitation (515-565) | Emission  (550-660) | Excitation (460-500) | Emission (510-560) |
| PI | 490-580 | 590-690 | ++ | ++ | + | - |
| Nile red | 500-550 | 560-690 | ++ | ++ | + | + |

Two filter sets were used for observation. Filter 1 revealed both PI (polysaccharides) and Nile red (hydrocarbons), while filter 2 revealed Nile red only, meaning that hydrocarbons appeared under both filter sets, while polysaccharides appeared only under filter 1. ImageJ software was used to merge images obtained using both filters in such a way that hydrocarbons appear in green and polysaccharides in shining red/orange. Hydrocarbons appeared in a shade of green with a low wavelength in images obtained under filter 1, and in dim red/orange under those under filter 2. Intensities of both images mixed by imageJ led to a high wavelength green. Polysaccharides colors were not affected by superposition, as they were detected by 1 filter only. However, when hydrocarbons and polysaccharides were overlapped on a zone of an image, the resulting color was a mix between high wavelength green (hydrocarbons) and a shining red/orange (polysaccharides). Consequently, the color at those locations appeared as yellow.

# 2. Results:

## Real-time observation during nsPEF treatment under microscope

Figure S2 provides more images than what has been shown in Fig. 3, performed during application of 500 pulses with 10 Hz repletion rate and electric field of 144 kV/cm.

The same experiment was carried out using a 3 mm gap between the copper electrodes, resulting in a lower electric field of 43.3 kV/cm. The result is shown in Fig. S3. Only a few cells (shown by arrows) separated from the colony when 500 shots of 43.3 kV/cm were applied. This corresponds with the results of electroporation cuvette and quantification of extraction, in which conditions of algae treatment correspond to an expended energy near 20 J in cuvette. According to the 3D graph of Fig. 6, 20 J at 43.3 kV/cm of nsPEF treatment is insufficient for extraction.

| 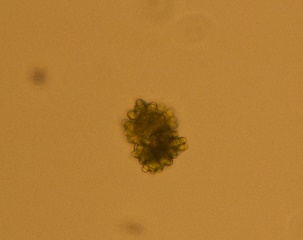  (a) | 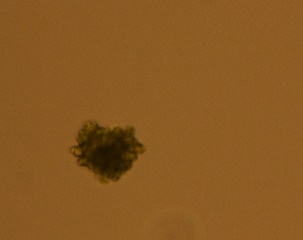  (b) | 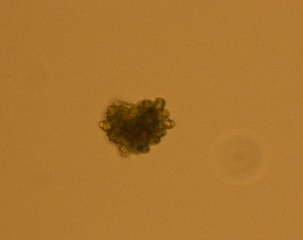  (c) | 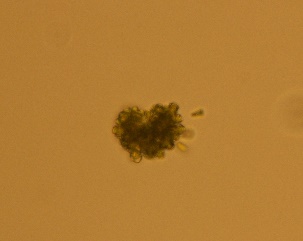  (d) |
| --- | --- | --- | --- |
|  |  |  |  |
| 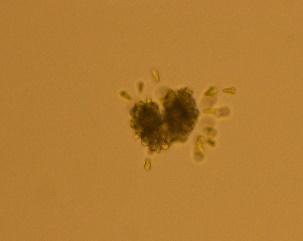  (e) | 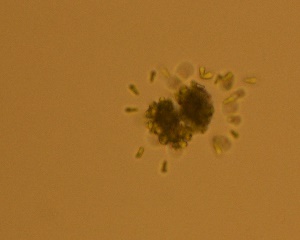  (f) | 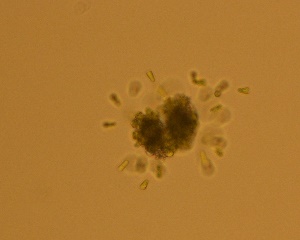  (g) | 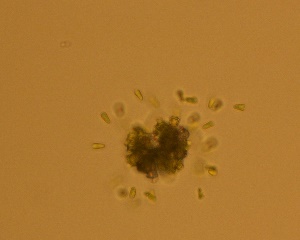  (h) |
|  |  |  |  |
| 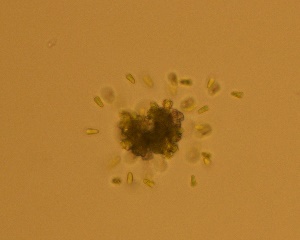  (i) | 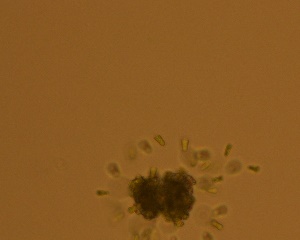  (j) | 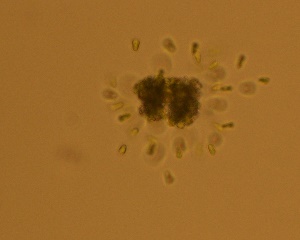  (k) | 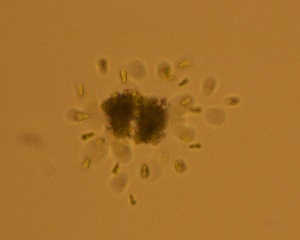  (l) |
|  |  |  |  |
| 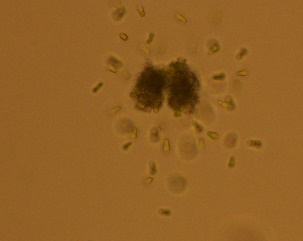  (m) | 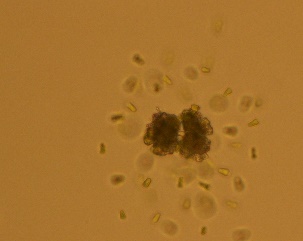  (n) | 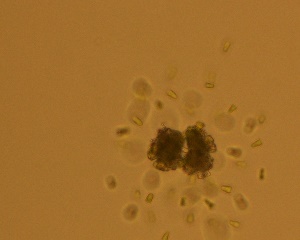  (o) | 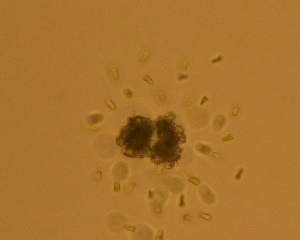  (p) |

**Fig. S2.** Real-time microscopic observation during treatment (500 pulses, 10 Hz, and 144 kV/cm) (magnification x20). (a)10 sec before treatment; (b) just before treatment; (c) after 100 pulses at 10 sec; (d) after 200 pulses at 20 sec; (e) after 300 pulses at 30 sec; (f) after 400 pulses at 40 sec; (g) after 500 pulses at 50 sec; (h) after 60 sec; (i) after 70 sec; (j) after 80 sec; (k) after 90 sec; (l) after 100 sec; (m) after 200 sec; (n) after 300 sec; (o) after 400 sec; (p) after 500 sec.

## 2.2 Comparison of the treated and sham treated colonies

Movie “additional file 2” shows a colony treated with nsPEFs (500 pulses at 10 Hz pulse repletion rate with 64 kV/cm equivalent). The sample colony was taken from a treated 2 mm gap cuvette, processed the same as described in section S-1.3. For comparison, movie “additional file 3” shows a colony underwent the same process as movie “additional file 2”, except the nsPEF pulses (sham treated). Movies were recorded with 6 frame/min speed (10 sec inter-frame timing), this gave us the possibility to increase magnification and refocus the microscope between the frames. As seen in fluorescent images of Fig. 2, in movies file-2 and file-3 also, due to pressure of the cover glass, hydrocarbons were pushed out of colonies. However, there is a major difference between movies file-2 and file-3; in movie file-2 (nsPEF treated) there is no more algae cell in the colony at the end of the movie; while in movie file-3 (sham treated), all algae cells in the colony are visible at the end of the movie.

| 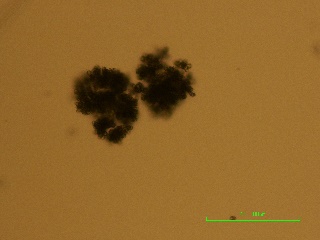  (a) | 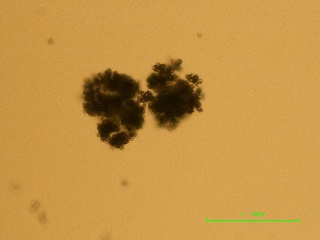  (b) | 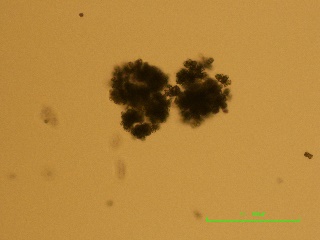  (c) | 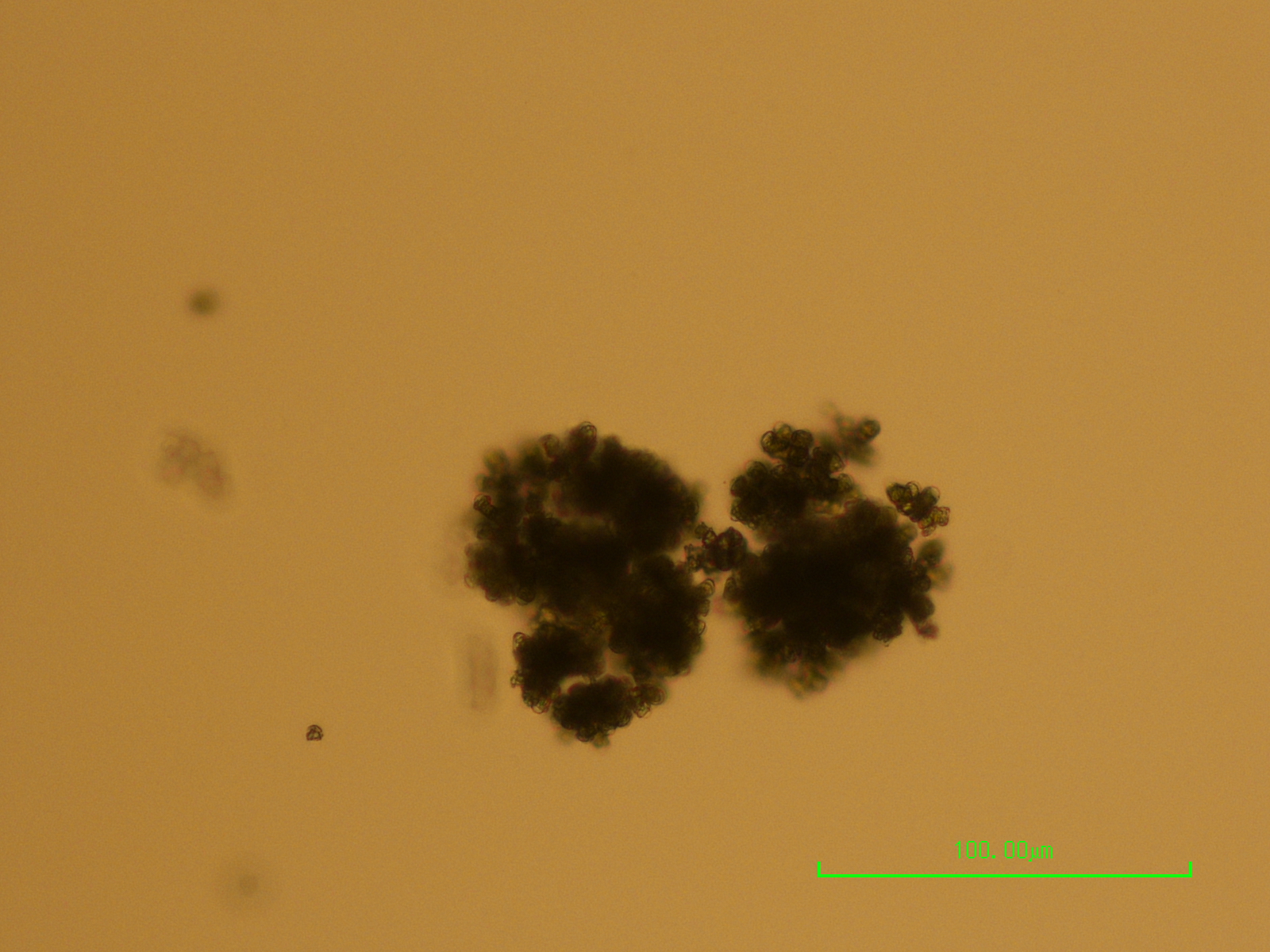 (d) |
| --- | --- | --- | --- |
|  |  |  |  |
| 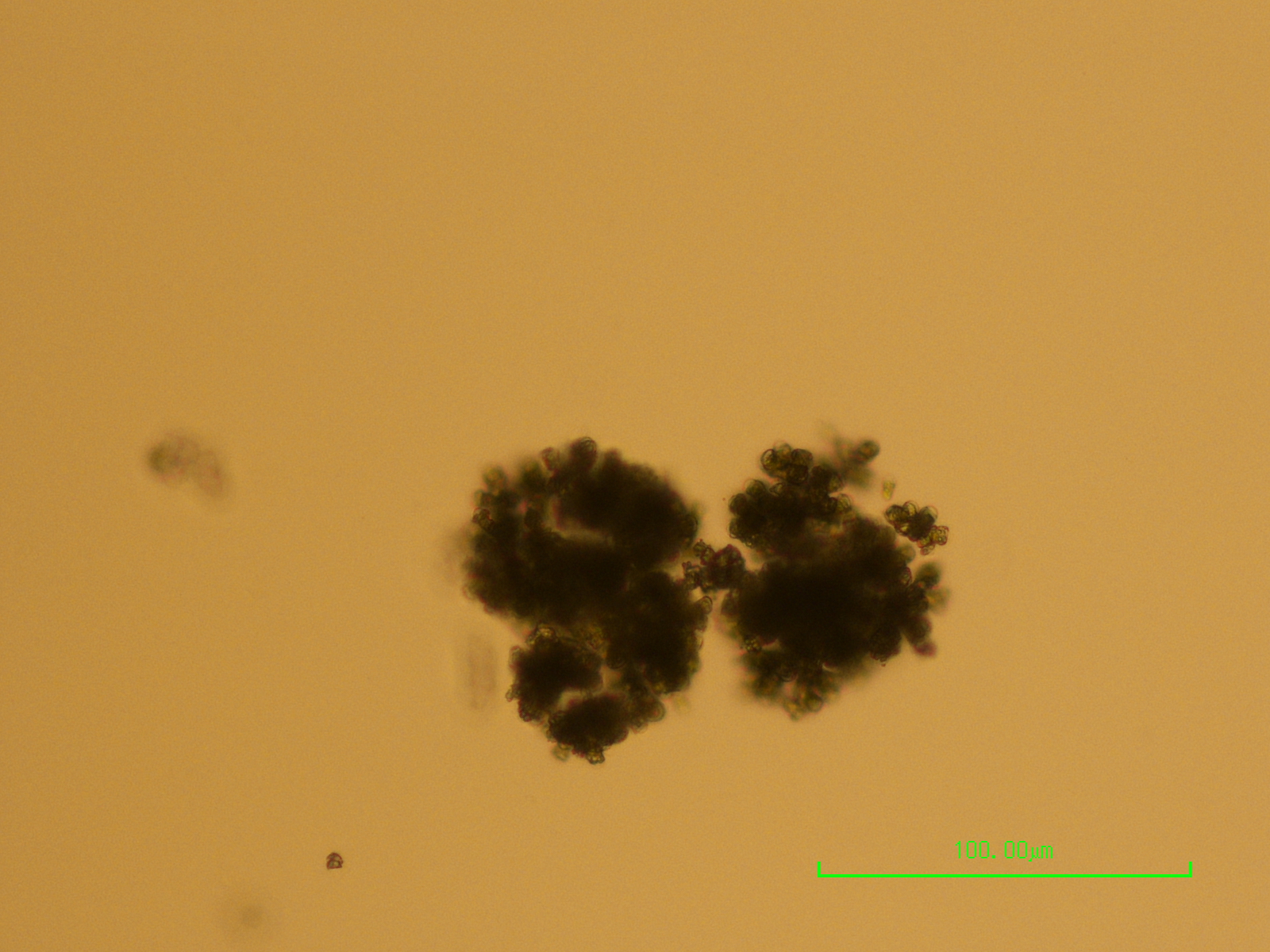 (e) | 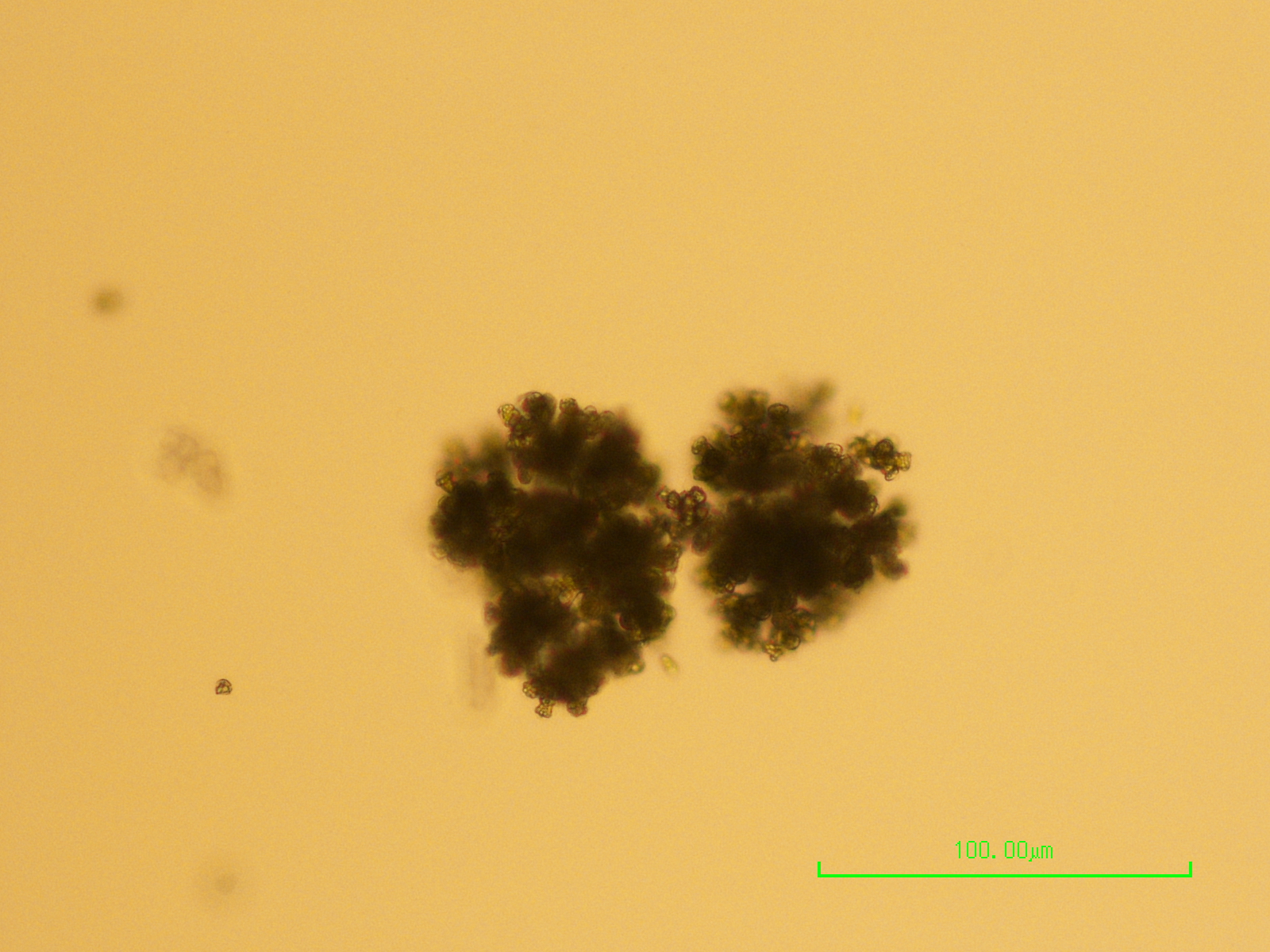 (f) | 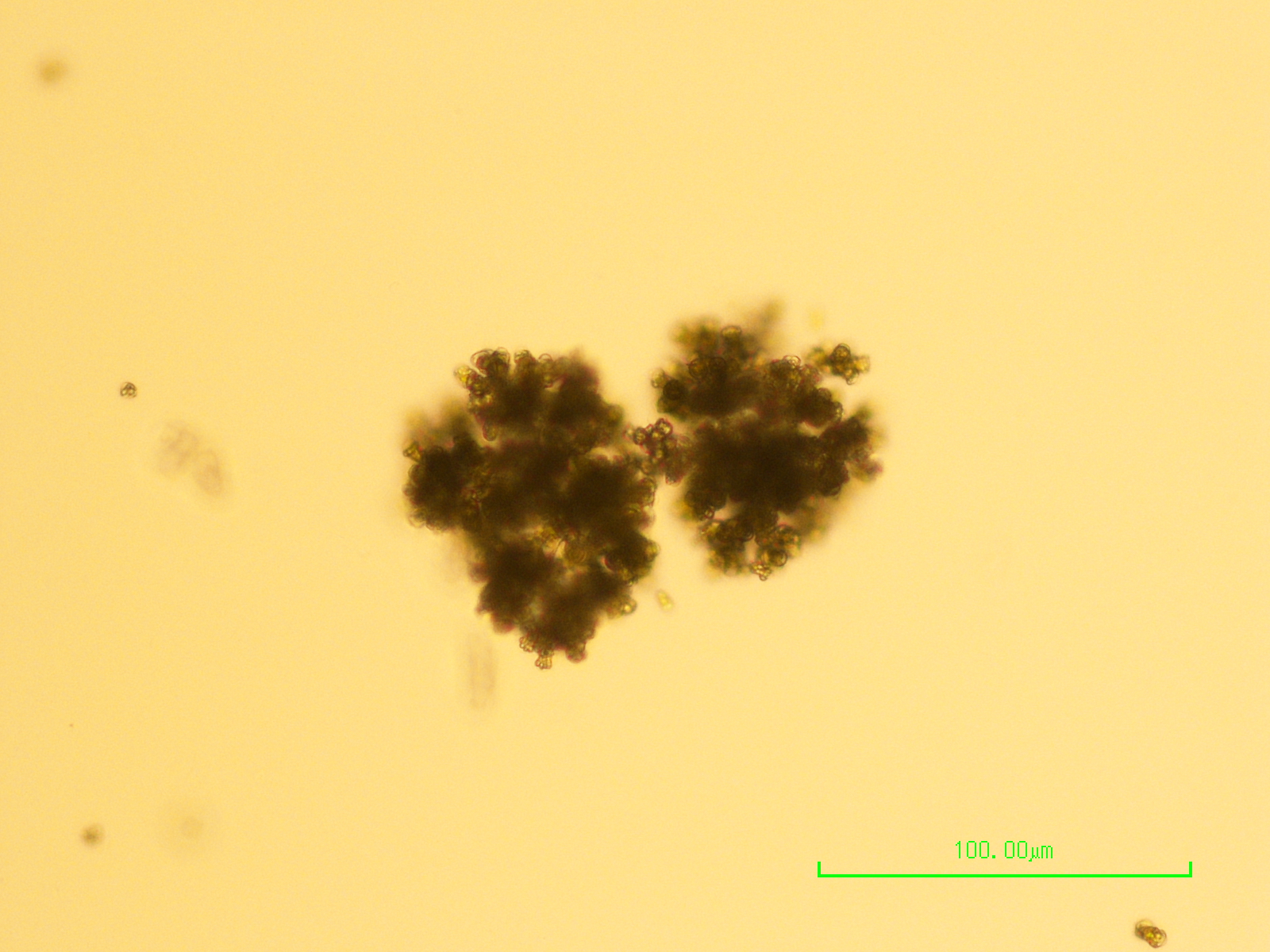 (g) | 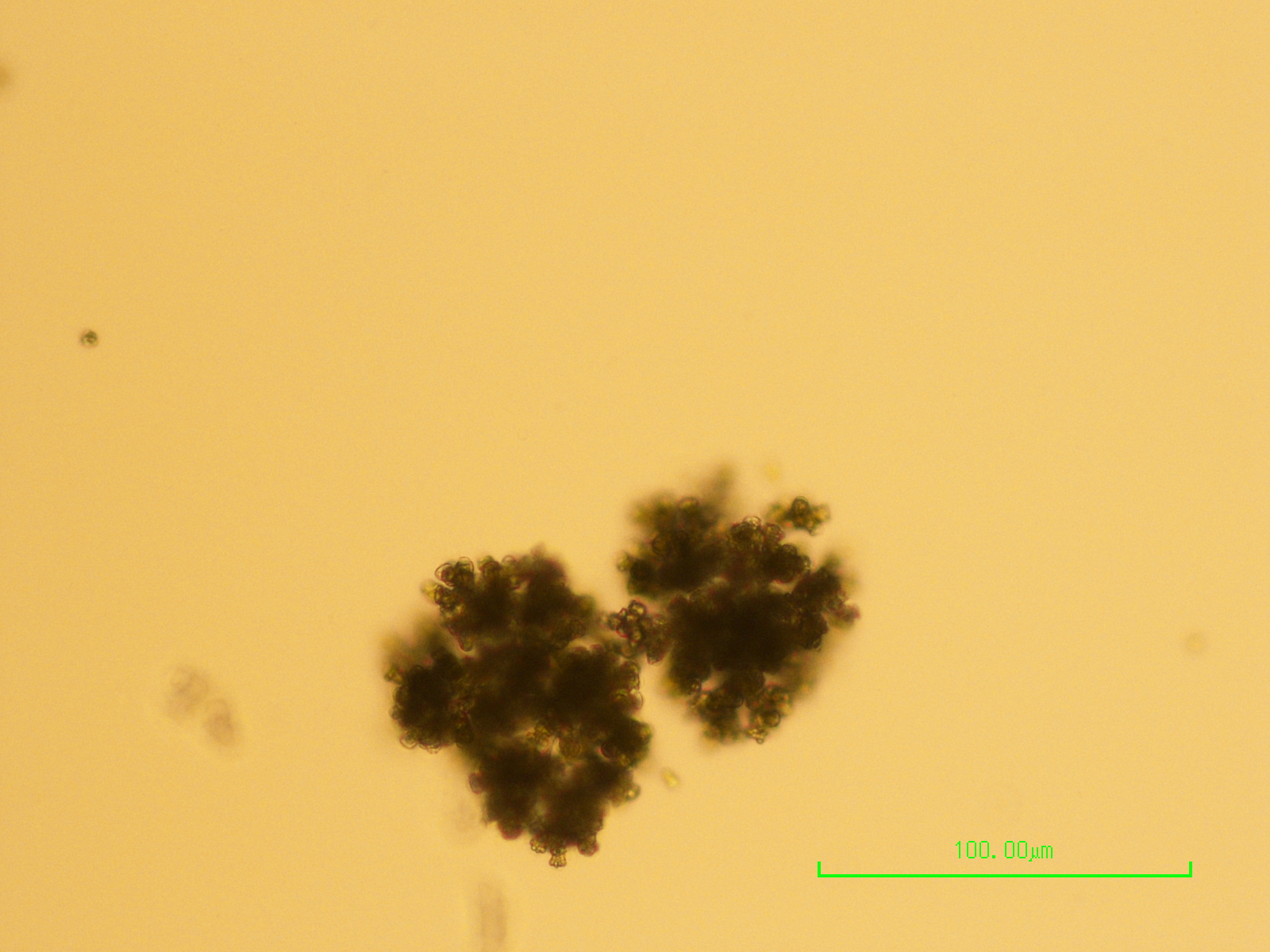 (h) |
|  |  |  |  |
| 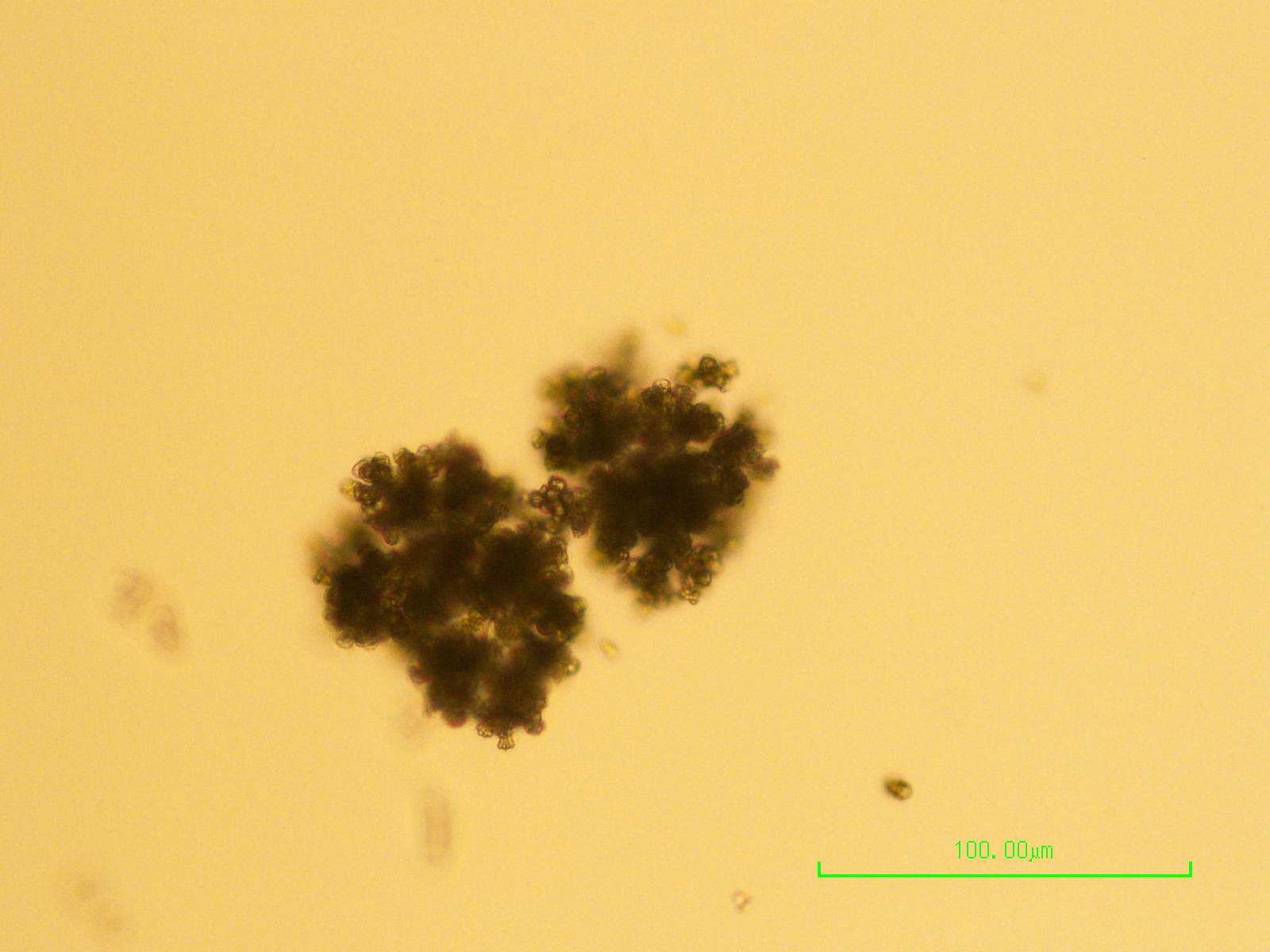 (i) | 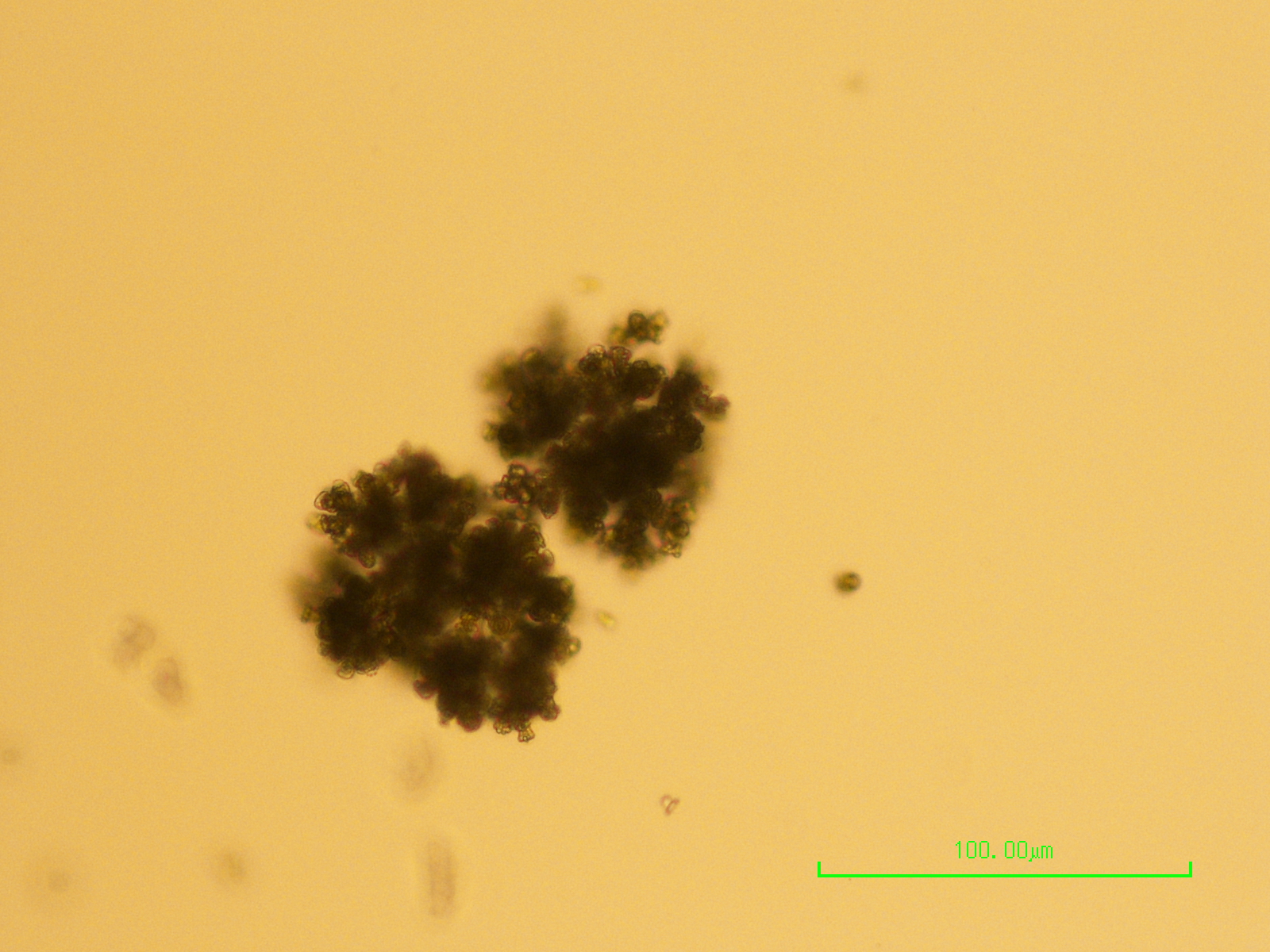 (j) | 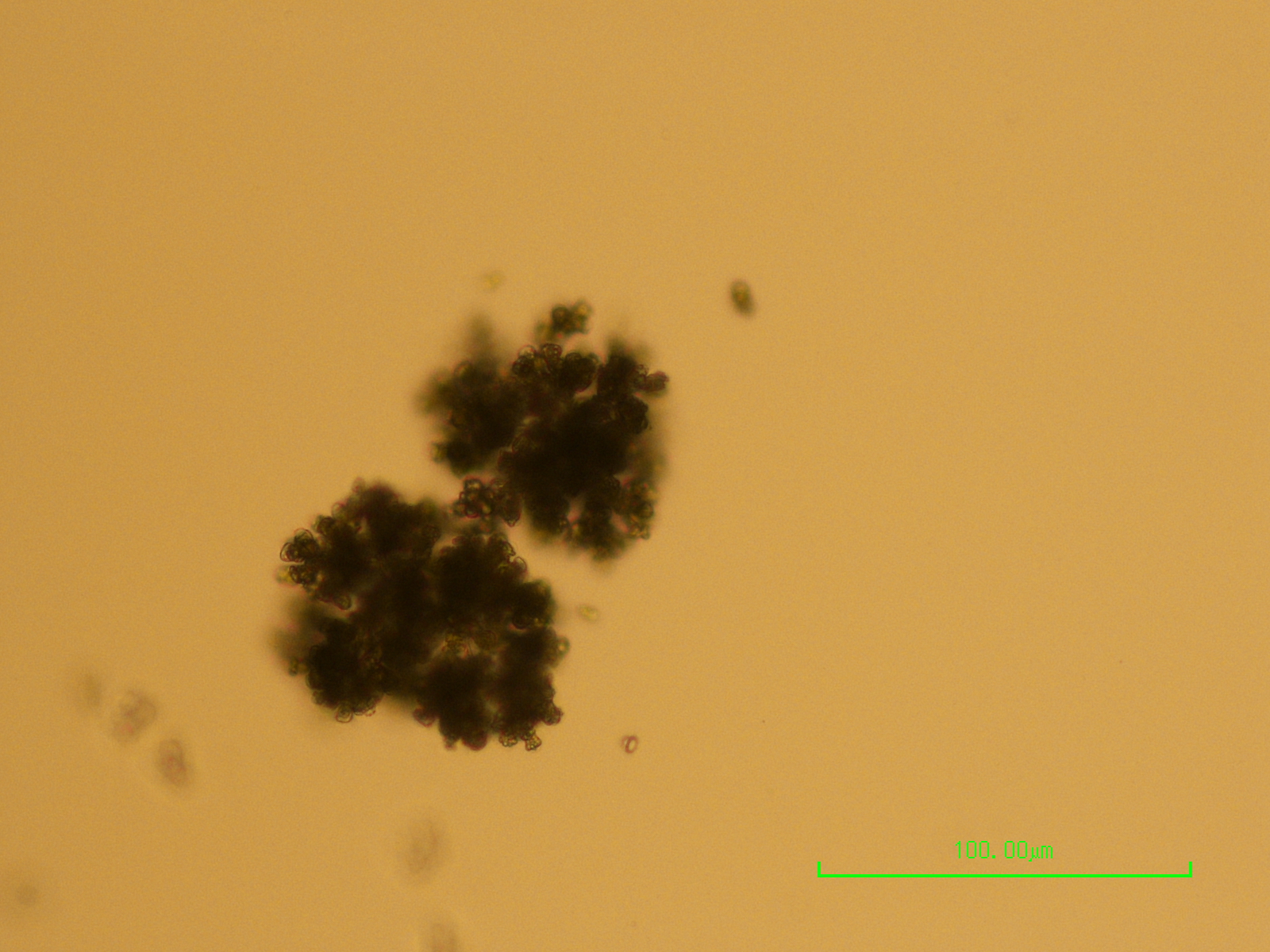 (k) | 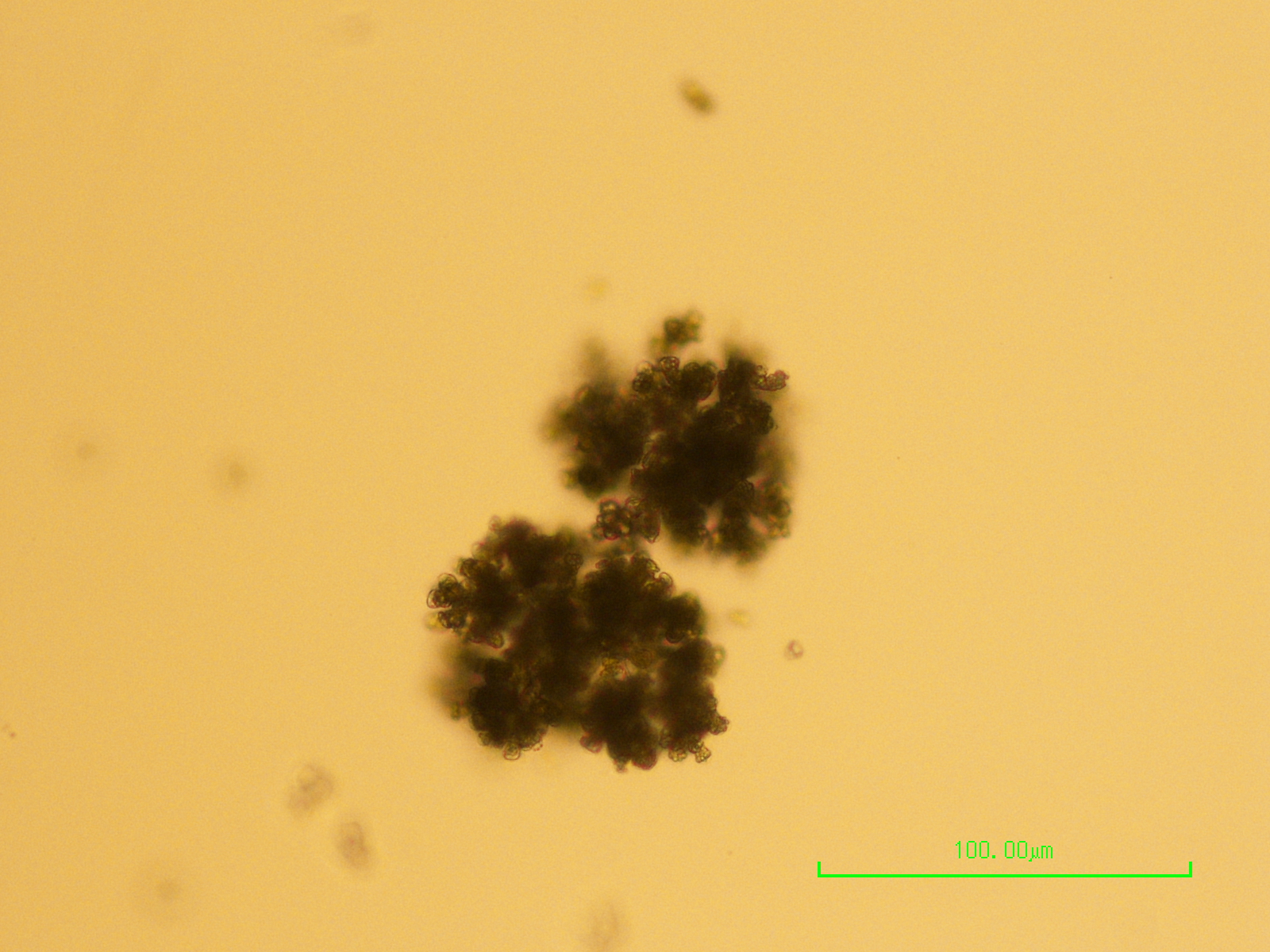 (l) |
|  |  |  |  |
| 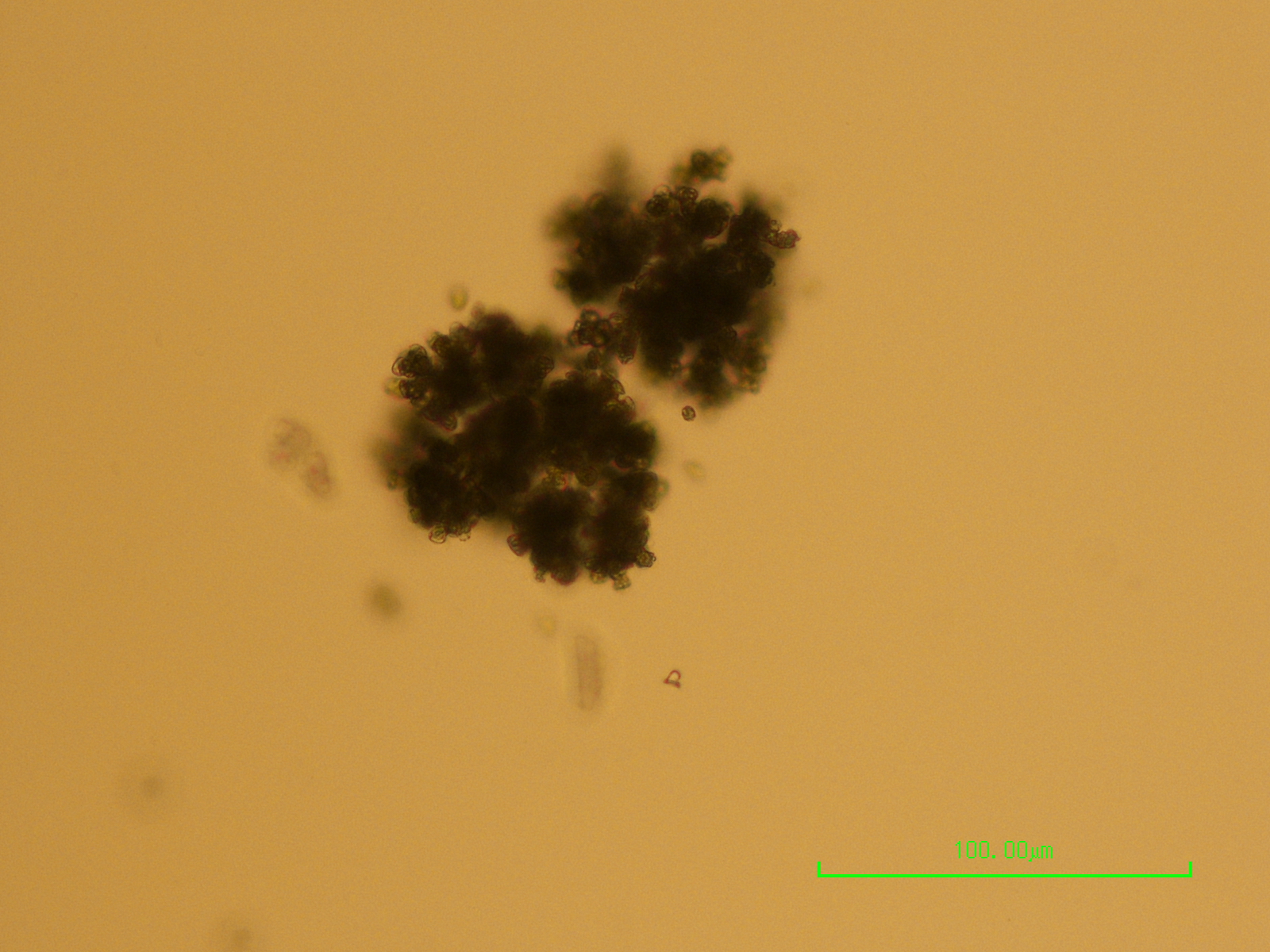 (m) | 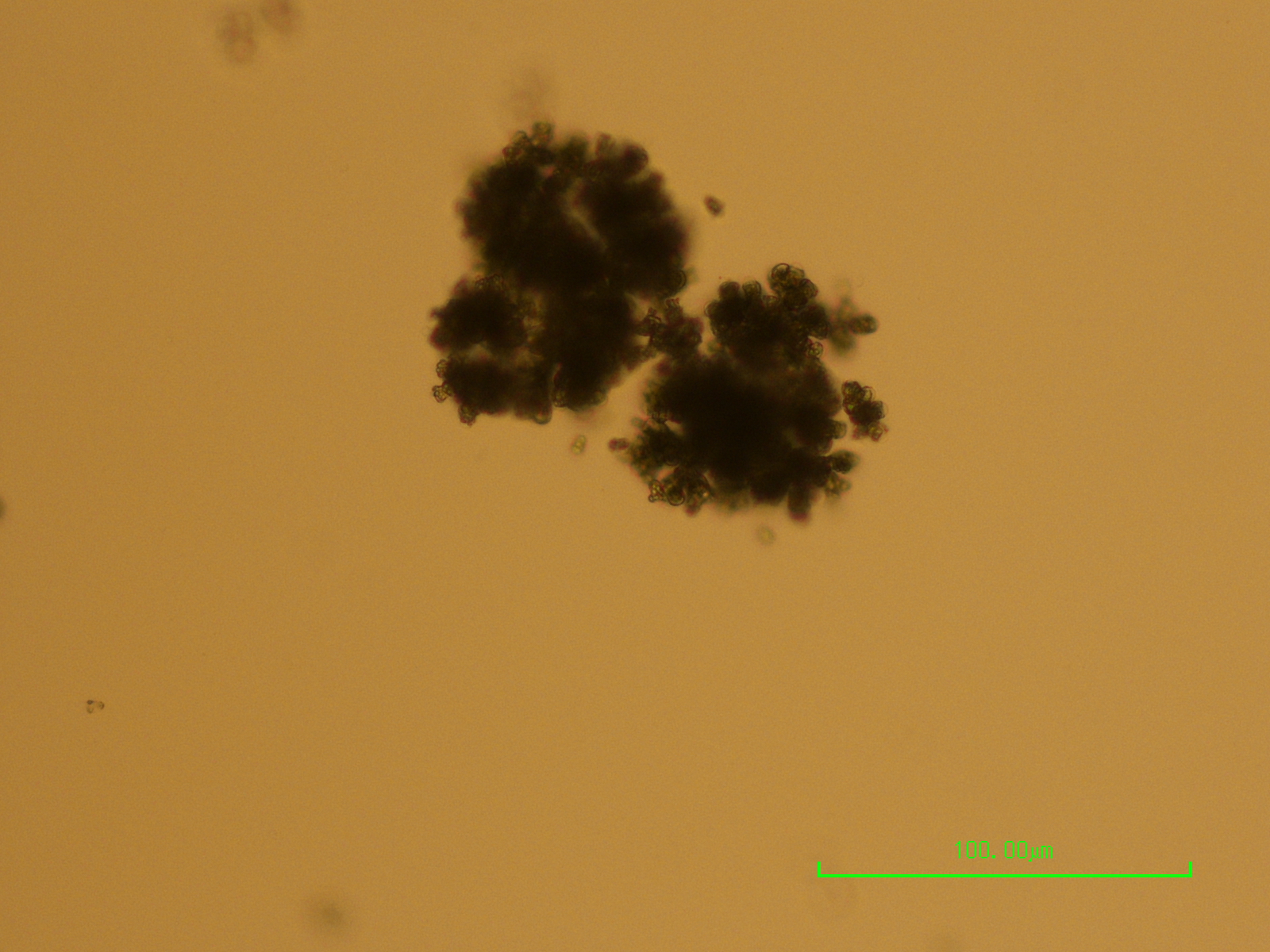 (n) | 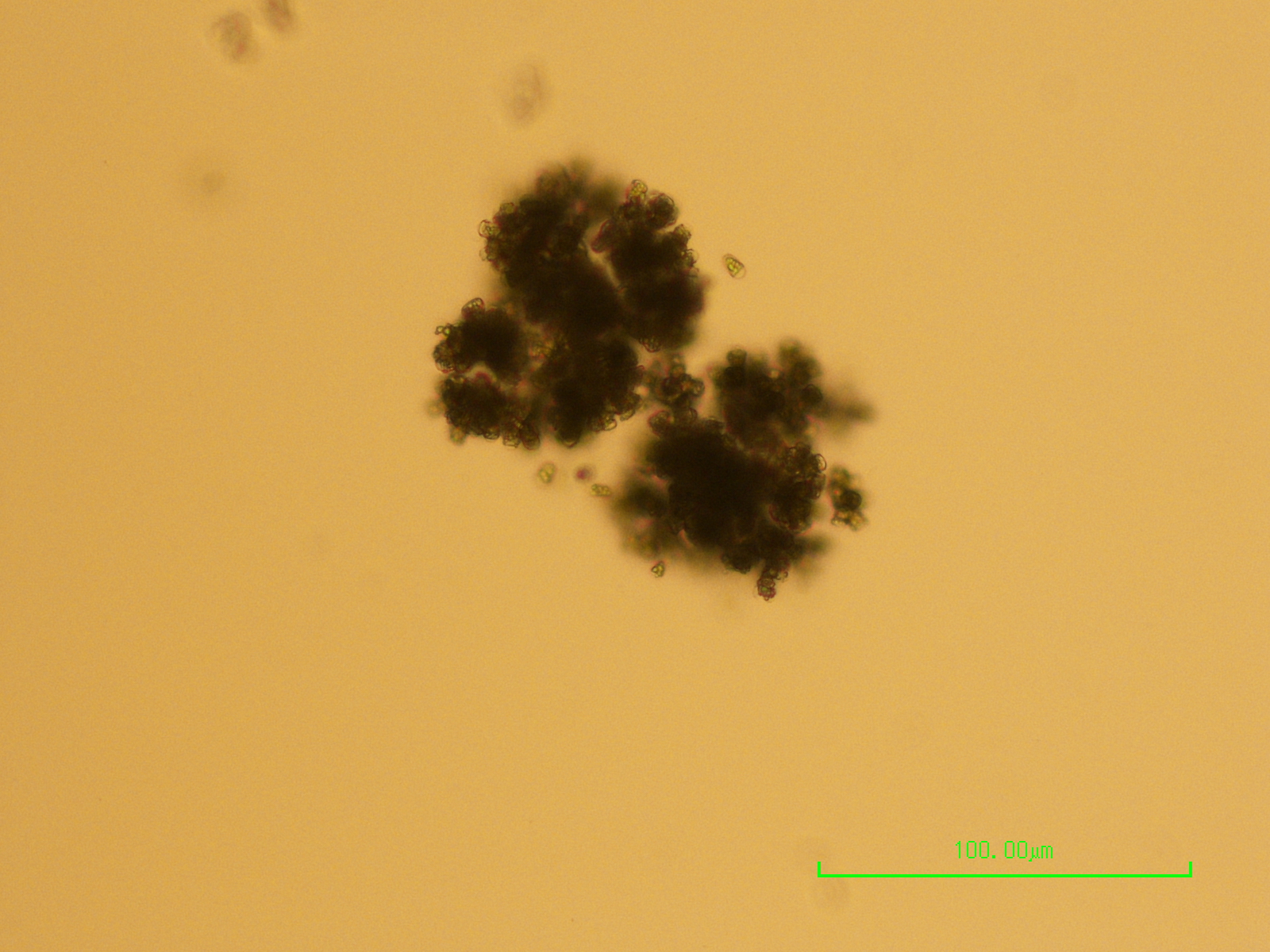 (o) |  |

**Fig. S3.** Real-time microscopic observation during treatment (500 pulses, 10 Hz, and 43.3 kV/cm) (magnification x20). (a)10 sec before treatment; (b) just before treatment; (c) after 100 pulses at 10 sec; (d) after 200 pulses at 20 sec; (e) after 300 pulses at 30 sec; (f) after 400 pulses at 40 sec; (g) after 500 pulses at 50 sec; (h) after 60 sec; (i) after 70 sec; (j) after 80 sec; (k) after 90 sec; (l) after 100 sec; (m) after 200 sec; (n) after 300 sec; (o) after 400 sec.

## Macroscopic evaluation

To observe the electric field effect on algae behavior at a macroscopic scale, all 10 cuvettes connected to the generator were used to treat a large volume. The experiment was repeated to obtain 50 mL of algae treated by 500 pulses at 10 Hz pulse repletion rate at 64 kV/cm.

|  | 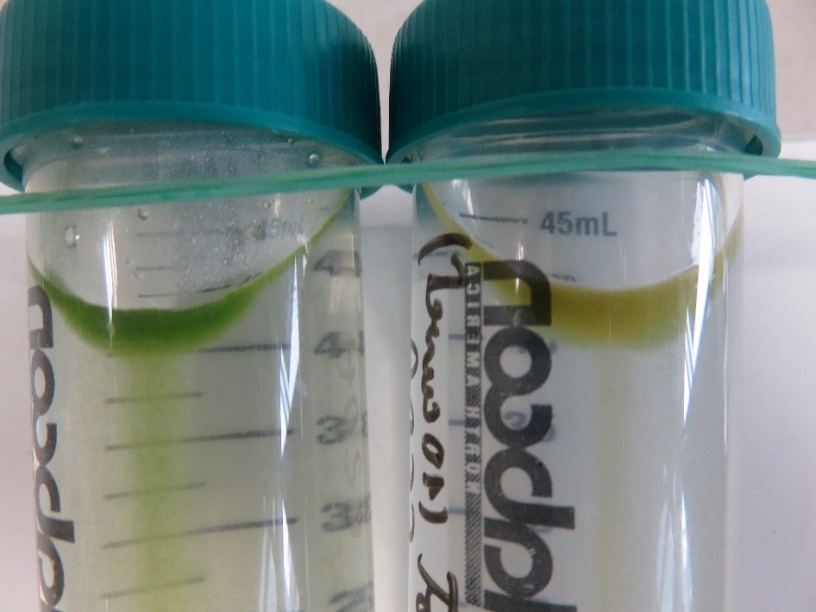  Control Treated  (a) (b) |
| --- | --- |
|  | 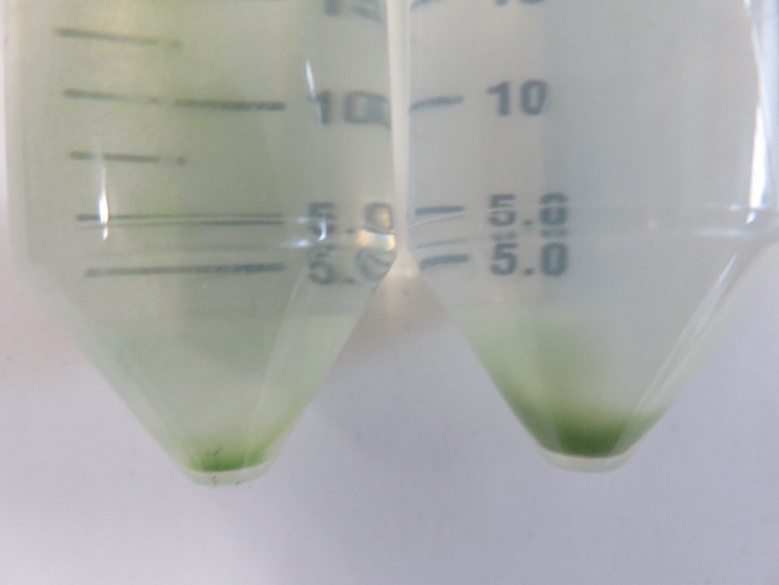  Control Treated  (c) (d) |

***Fig. S4.*** *Supernatant of (a) control, (b) treated; and pellet (sediment) of (c) control, (d) treated samples; both after centrifuging for 15 min at 2,000 g*

Figure S4 shows a comparison between control and nsPEF treated results. The matrix contain hydrocarbons and polysaccharides with a color mainly yellow/brown. However, the usual color of algae colonies is green due to the chloroplast inside cells containing green pigment, chlorophyll. The supernatant is green in the control sample but yellow/brown in the treated sample, suggesting that treated supernatant is free of cells. The amount of sediment pellet in the treated sample is much more than in the control sample, because cells separated from colonies by nsPEF settled at the bottom of the test tube. The observation confirm the microscopic result that nsPEF separates cells from matrix.

## 2.4 Thin layer chromatography

The results of thin layer chromatography are shown in Fig. S5. With solvent for polar lipids (Fig. S5-a,b), all spots were migrated. As algae samples were soluble in a sufficiently strong solvent, this confirms they were mainly composed of lipids (Fig. S5-a,b). Moreover, only samples of oil extracted by nsPEF (spot-3) and phosphatidylcholine (polar lipid) (spot-4) were not completely eluted, suggesting that algae extracts were polar lipids. With solvent for apolar lipids (Fig. S5-c,d), squalene (spot-6) were completely eluted to the solvent front; phosphatidylcholine (spot-4) stayed at the initial position; tristearine (spot-5) was not visible on direct UV revelation, but appeared clearly migrated on the middle of the layer after Rhodamine-B coloration; oil extracted by nsPEF (spot-2), oil extracted by cyclohexane and heat treatment (spot-3), and control (spot-7) showed small stains near the solvent front but mainly stain stayed at the initial position, like phosphatidylcholine behavior. The results of both polar and apolar solvents confirmed that the main composition of algae extract were polar lipids.

| Direct UV light (contrast enhanced by ImageJ) | Rhodamine-B coloration |
| --- | --- |
| Migration using; chloroform : acetone : methanol : acetic acid : water (as solvent for polar lipids) | |
| 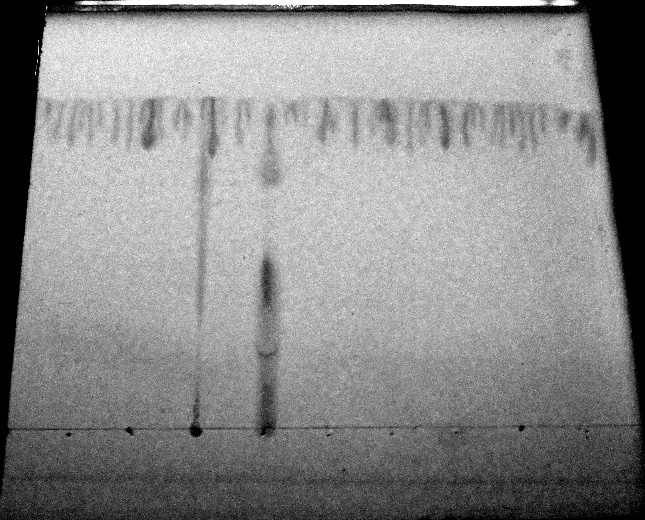  1 2 3 4 5 6 7  (a) | 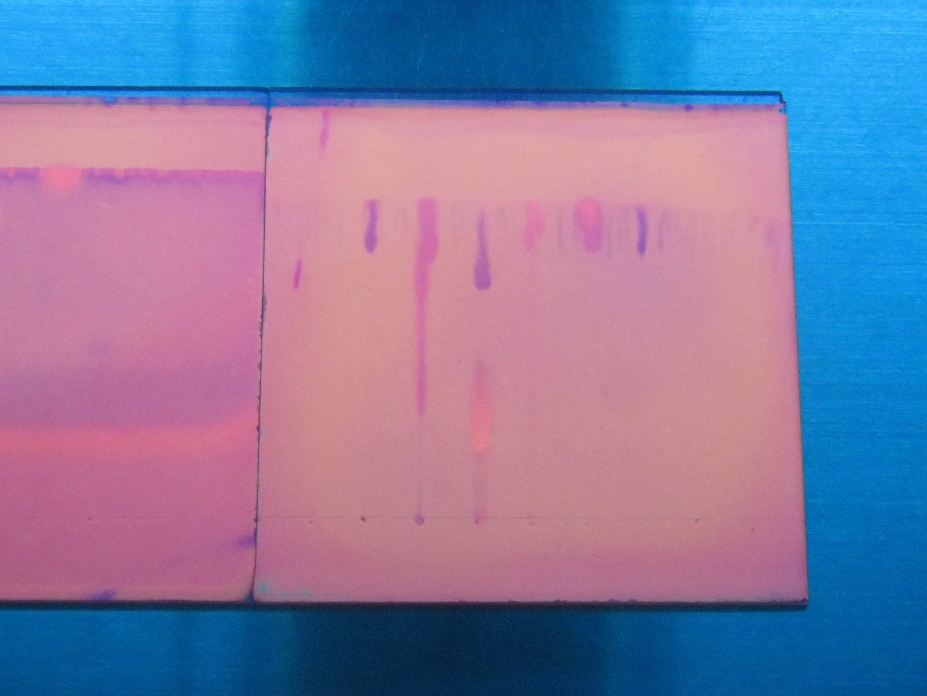  1 2 3 4 5 6 7  (b) |
| Migration using; hexane : diethyl ether : acetic acid (as solvent for apolar lipids) | |
| 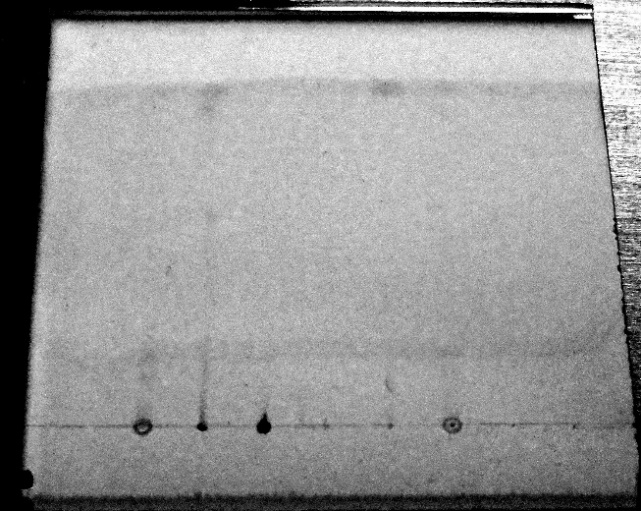  1 2 3 4 5 6 7  (c) | 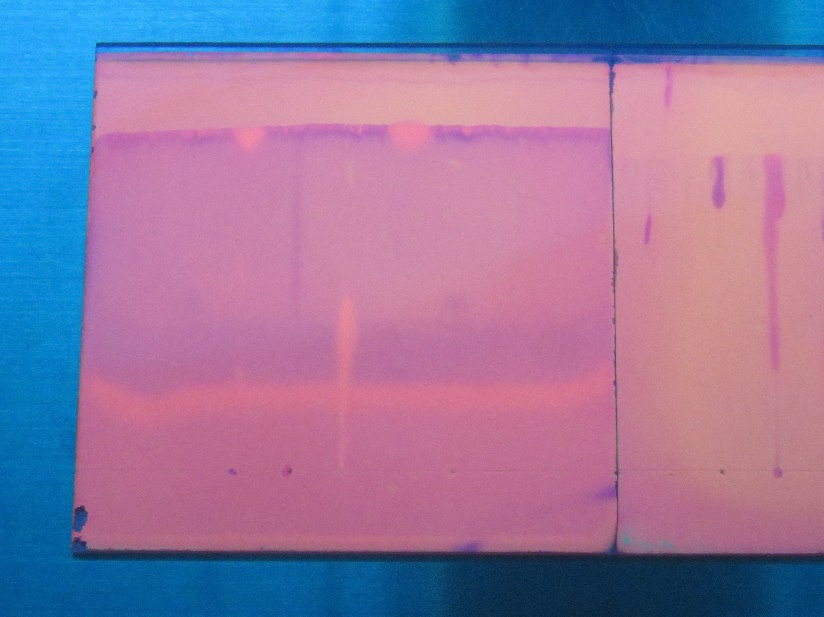  1 2 3 4 5 6 7  (d) |

**Fig. S5.** Thin layer chromatography with solvents for (a-b) polar and (c-d) apolar lipids.

spot-2:oil extracted by nsPEF, spot-3:oil extracted by cyclohexane and heat treatment at 80°^C^,

spot-4:phosphatidylcholine (phospholipid), spot-5:tristearine (triglyceride), spot- 6:squalene, spot-7:oil extracted without nsPEF (control)

## 2.5 Image analysis

Figure S6 shows three samples treated with different numbers of pulses at 10 Hz pulse repetition rate with 21.5 kV/cm, 39 kV/cm, and 64 kV/cm electric fields. Figure S6 represents examples of images which were used to determine the extraction efficiency. For 39 kV/cm and 64 kV/cm samples, the green supernatant turns yellow by increasing the shot number. Photos were analyzed with the open source software “ImageJ”.

Figure S6 clearly reassures that there is an electric field threshold for the extraction; extraction was not possible even with 10,000 shots at low electric field of 21.5 kV/cm (supernatant of control and 10,000 shots are the same for 21.5 kV/cm samples).

| 10Hz, 21.5 kV/cm | 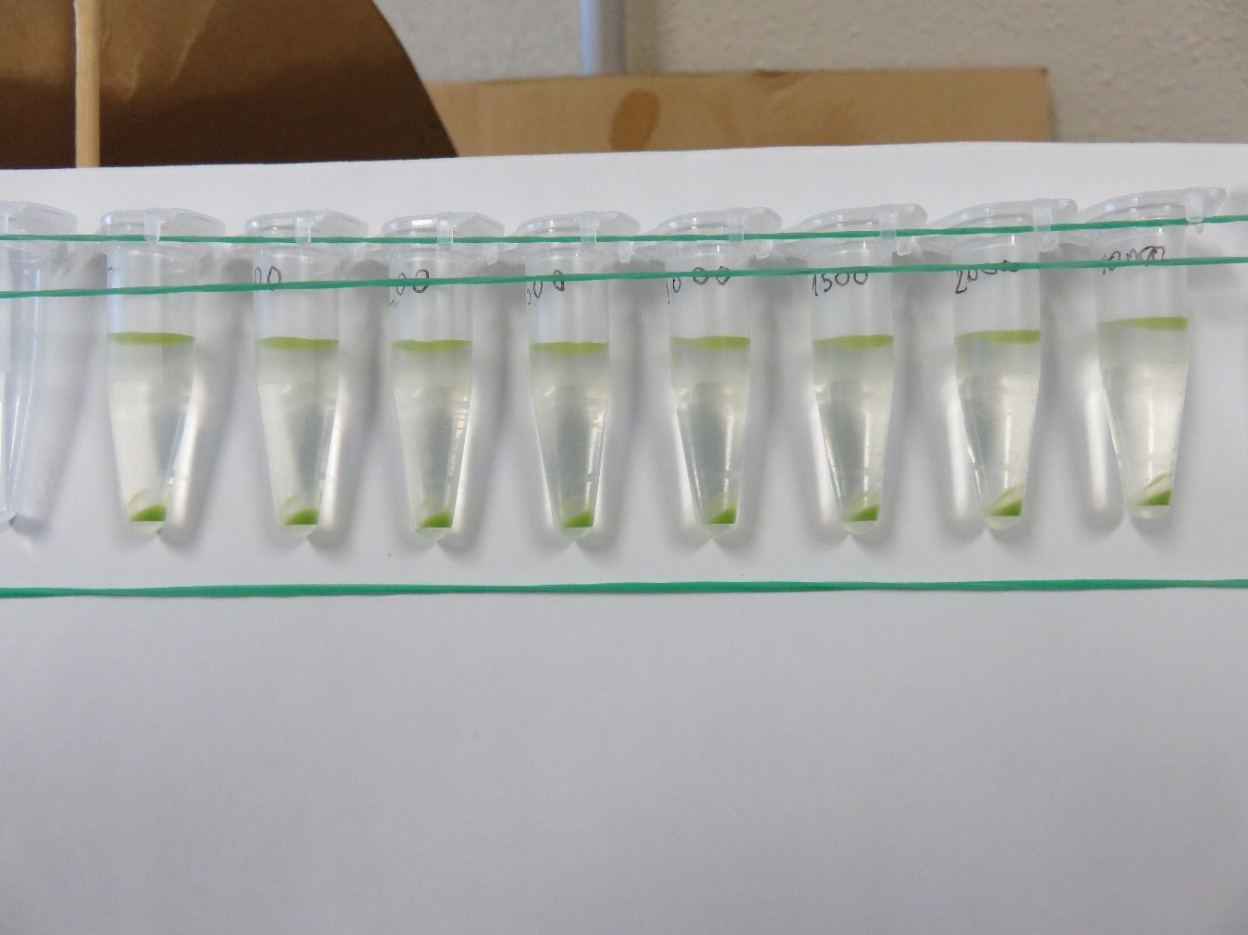  0 100 200 500 1 000 1 500 2 000 10 000 |
| --- | --- |
| 10Hz, 39 kV/cm: | 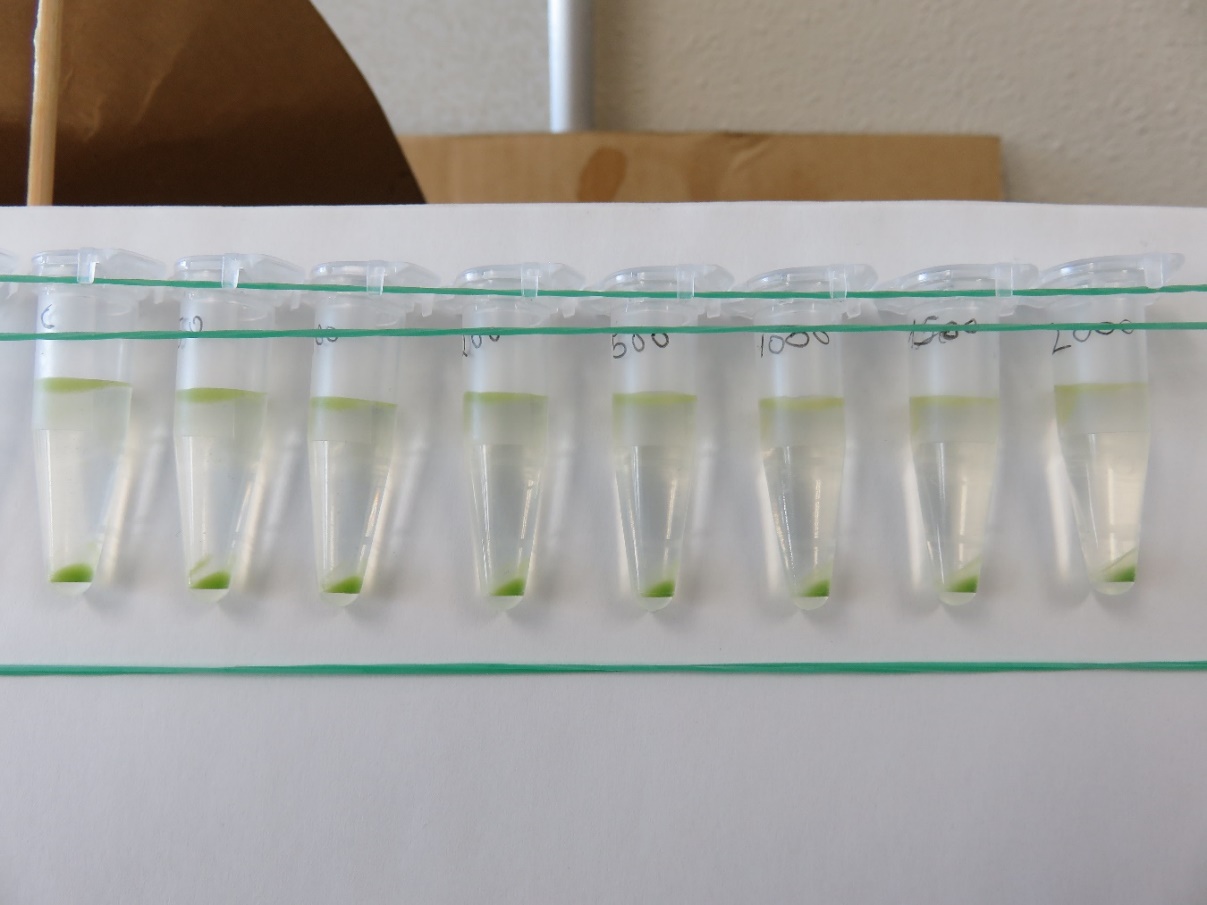  0 50 100 200 500 1 000 1 500 2 000 |
| 10Hz, 64 kV/cm: | 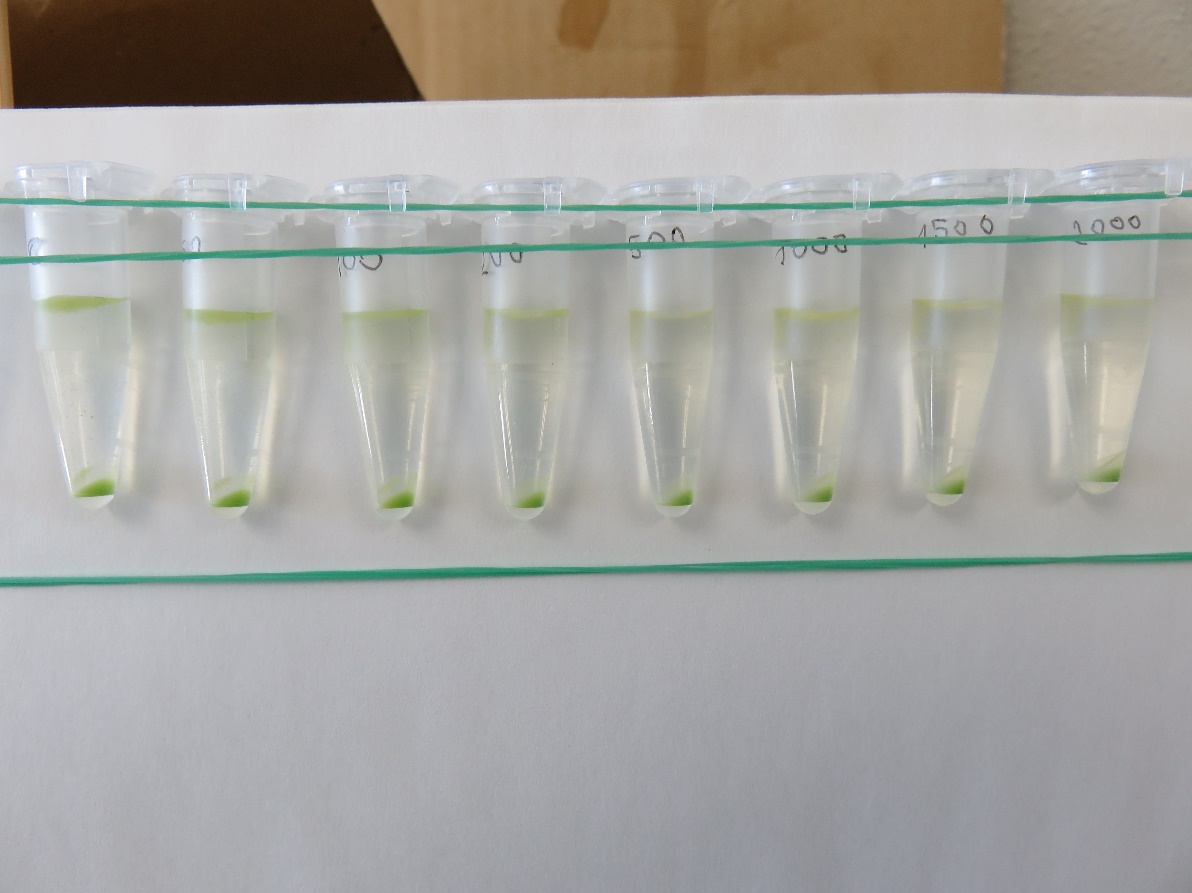  0 50 100 200 500 1 000 1 500 2 000 |

***Fig. S6.*** *Images of samples treated with 21.5 kV/cm, 39kV/cm, and 64 kV/cm electric fields at 10 Hz pulse repetition rate with different number of pulses.*

## 2.6 Schematic representation of *botryococcus braunii* growth

Figure S7 is drawn based on the fluorescence microscopy observations (Fig. 2), which explains *botryococcus braunii* growth method leading to dichotomous branching of the matrix.

**Fig. S7.** Putative mechanism of Botryococcus braunii colony growth starting from single cell.

# Supplementary References

1. Kang, D.K., Hosseini, H., Shiraishi, E., Yamanaka, M. & Akiyama, H. Single nanosecond pulsed electric field effects on embryonic development of medaka fish. *IEEE Trans. Plasma. Sci.* **40**, 2379–2387 (2012).
2. Shiraishi, E., Hosseini, H., Kang, D.K., Kitano, T. & Akiyama, H. Nanosecond pulsed electric field suppresses germ cell proliferation and meiosis through blocking retinoic acid signaling in medaka (Oryzias latipes). *PLoS ONE* **8**, e70670 (2013).
3. Huang, G.H, Chen, G. & Chen, F., Rapid screening method for lipid production in alga based on Nile red fluorescence. *Biomass Bioenergy* **33**, 1386–1392 (2009).
4. Weiss, T.L., Roth, R., Goodson, C., Vitha, S., Black, I., Azadi, P., et al. Colony organization in the green alga Botryococcus braunii (race B) is specified by a complex extracellular matrix. *Eukaryotic Cell* **11**, 1424–40 (2012).
